# Longitudinal assessment of reactivity and affinity profile of anti-Jo1 autoantibodies to distinct HisRS domains and a splice variant in a cohort of patients with myositis and anti-synthetase syndrome

**DOI:** 10.1186/s13075-022-02745-6

**Published:** 2022-03-02

**Authors:** Antonella Notarnicola, Charlotta Preger, Susanna L. Lundström, Nuria Renard, Edvard Wigren, Eveline Van Gompel, Angeles S. Galindo-Feria, Helena Persson, Maryam Fathi, Johan Grunewald, Per-Johan Jakobsson, Susanne Gräslund, Ingrid E. Lundberg, Cátia Fernandes-Cerqueira

**Affiliations:** 1 Division of Rheumatology, Department of Medicine, Karolinska University Hospital, Karolinska Institutet, SE-171 64, Solna, Stockholm, Sweden; 2 Center for Molecular Medicine, Karolinska Institutet, Stockholm, Sweden; 3 Structural Genomics Consortium, Toronto, Canada; 4 Division of Physiological Chemistry I, Department of Medical Biochemistry and Biophysics, Karolinska Institutet, Solnavägen 9, SE-171 77 Stockholm, Sweden; 5 Laboratory of Tissue Homeostasis and Disease, Skeletal Biology and Engineering Research Center, KULeuven, Leuven, Belgium; 6 Science for Life Laboratory, Drug Discovery and Development, Stockholm, Sweden; 7 School of Engineering Sciences in Chemistry, Biotechnology and Health, Royal Institute of Technology (KTH), Stockholm, Sweden; 8 Department of Respiratory Medicine and Allergy, J7:30, Bioclinicum, Karolinska University Hospital, Karolinska Institutet, SE-171 76 Stockholm, Sweden; 9 4Dcell, 14 rue de la Beaune, 93100 Montreuil, France

**Keywords:** Anti-Jo1, HisRS, Longitudinal samples, ILD, Autoantibodies, Affinity, Reactivity, BALF, Idiopathic inflammatory myopathies, Anti-synthetase syndrome

## Abstract

**Background:**

To address the reactivity and affinity against histidyl-transfer RNA synthetase (HisRS) autoantigen of anti-Jo1 autoantibodies from serum and bronchoalveolar lavage fluid (BALF) in patients with idiopathic inflammatory myopathies/anti-synthetase syndrome (IIM/ASSD). To investigate the associations between the reactivity profile and clinical data over time.

**Methods:**

Samples and clinical data were obtained from (i) 25 anti-Jo1^+^ patients (19 sera with 16 longitudinal samples and 6 BALF/matching sera at diagnosis), (ii) 29 anti-Jo1^−^ patients (25 sera and 4 BALF/matching sera at diagnosis), and (iii) 27 age/gender-matched healthy controls (24 sera and 3 BALF/matching sera). Reactivity towards HisRS full-length (HisRS-FL), three HisRS domains (WHEP, antigen binding domain (ABD), and catalytic domain (CD)), and the HisRS splice variant (SV) was tested. Anti-Jo1 IgG reactivity was evaluated by ELISA and western blot using IgG purified from serum by affinity chromatography. In paired serum-BALF, anti-Jo1 IgG and IgA reactivity was analyzed by ELISA. Autoantibody affinity was measured by surface plasmon resonance using IgG purified from sera. Correlations between autoantibody reactivity and clinical data were evaluated at diagnosis and longitudinally.

**Results:**

Anti-Jo1 IgG from serum and BALF bound HisRS-FL, WHEP, and SV with high reactivity at the time of diagnosis and recognized both conformation-dependent and conformation-independent HisRS epitopes. Anti-HisRS-FL IgG displayed high affinity early in the disease. At the time of IIM/ASSD diagnosis, the highest autoantibody levels against HisRS-FL were found in patients ever developing interstitial lung disease (ILD) and arthritis, but with less skin involvement. Moreover, the reactivity of anti-WHEP IgG in BALF correlated with poor pulmonary function.

Levels of autoantibodies against HisRS-FL, HisRS domains, and HisRS splice variant generally decreased over time. With some exceptions, longitudinal anti-HisRS-FL antibody levels changed in line with ILD activity.

**Conclusion:**

High levels and high-affinity anti-Jo1 autoantibodies towards HisRS-FL were found early in disease in sera and BALF. In combination with the correlation of anti-HisRS-FL antibody levels with ILD and ILD activity in longitudinal samples as well as of anti-WHEP IgG in BALF with poor pulmonary function, this supports the previously raised hypothesis that the lung might have a role in the immune reaction in anti-Jo1-positive patients.

**Supplementary Information:**

The online version contains supplementary material available at 10.1186/s13075-022-02745-6.

## Background

Idiopathic inflammatory myopathies (IIM) are rare autoimmune, chronic inflammatory diseases associated with high mortality and morbidity [1, 2]. A major IIM sub-group, termed anti-synthetase syndrome (ASSD), affects skeletal muscle, lung, joints, and skin and is characterized by the presence of autoantibodies that target aminoacyl transfer(t) RNA synthetases (aaRS) [3]. Anti-histidyl tRNA synthetase (HisRS) autoantibodies (anti-Jo1) are the most common anti-aaRS autoantibodies detected in 15–36% of IIM patients [4–6]. Remarkably, up to 90% of IIM/ASSD patients diagnosed with interstitial lung disease (ILD) have anti-Jo1 autoantibodies [7].

**Fig. 1:**
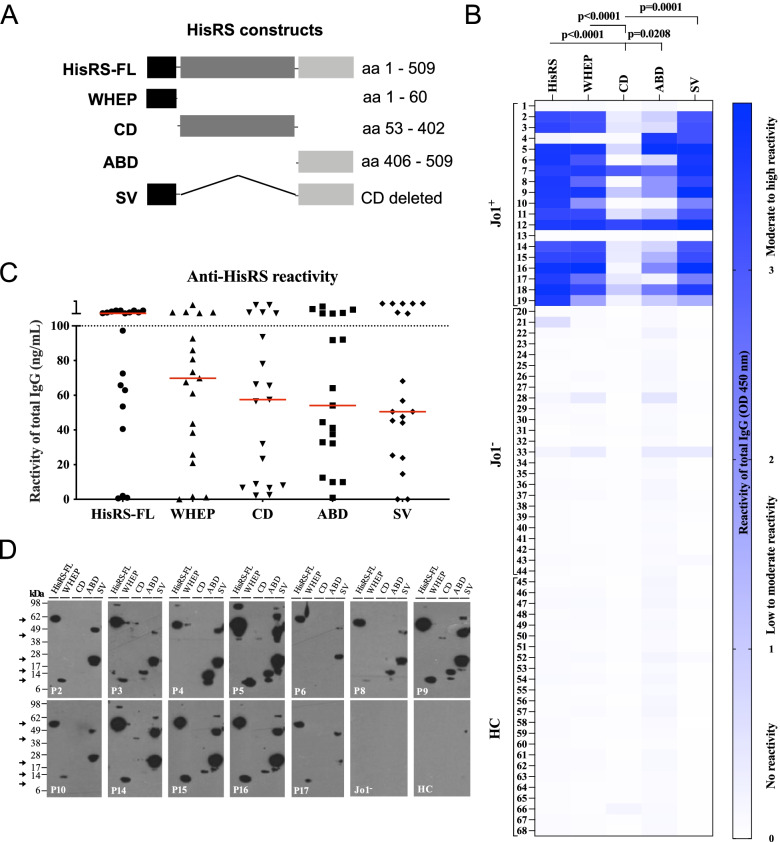
Anti-Jo1 autoantibodies display high reactivity against HisRS full-length/variant/domains at IIM/ASSD diagnosis. **A** Schematic figure of HisRS versions used in the experiments. **B** The reactivity towards HisRS-FL, WHEP, CD, ABD, and SV as conformational epitopes was measured by ELISA in IgG purified from serum of 19 anti-Jo1^+^, 25 anti-Jo1^−^, and 24 healthy controls (HC). High reactivity (OD 450 nm) corresponds to strong blue color. **C** Anti-HisRS reactivity (ng/mL concentration) of anti-Jo1^+^ total IgG against HisRS-FL, WHEP, CD, ABD, and SV. Antibody titers were calculated using a standard curve (Supplementary Fig. 3B) and titers were measured in the linear range between 5 and 100 ng/mL. Median values for each antigen are indicated by the red line. **D** Anti-Jo1 reactivity against denatured HisRS antigens was addressed by WB in IgG purified from serum of 12 representative anti-Jo1^+^, 1 anti-Jo1^−^, and 1 HC. Strong band intensity denotes higher anti-Jo1 reactivity. Arrows indicate the molecular weight of the different HisRS variants (Supplementary Table 2, Supplementary Fig. 1). Some patients also showed an additional band, corresponding to the HisRS dimer. Friedman’s test corrected for multiple comparisons using Dunn’s test in (**B**, **C**) was applied. In **B**, the calculations were done only on Jo1+ patients. *p* < 0.05 was assumed as significantly different

HisRS is a homodimeric protein composed of three domains, the WHEP domain located at the N-terminus, an internal catalytic domain (CD), and the anti-codon binding domain (ABD) at the C-terminal end (Fig. 1A) [8]. In 2012, a monomeric HisRS splice variant (SV) comprising the WHEP domain and the ABD (lacking the CD) was discovered [9]. Later, an additional HisRS splice variant composed of the first 60 amino acids (WHEP domain itself) was described and found to be overexpressed in the lung compared to other human tissues [10, 11]. Both full-length HisRS (HisRS-FL) and the WHEP domain were shown, in vitro, to be secreted from the cytosol of different cell lines including lung and muscle cells into the extracellular environment [10]. In addition, HisRS was detected in serum from patients with IIM/ASSD and in healthy individuals [12]. Serum levels of HisRS protein were lower in patients with anti-Jo1 autoantibodies compared to patients with IIM/ASSD without anti-Jo1 autoantibodies and healthy individuals [12]. Previous studies have demonstrated that the anti-Jo1 response in myositis is directed towards several epitopes within the HisRS molecule, and particularly the WHEP domain [13–18]. However, these studies were performed using linker mutagenesis and restriction enzymes, or linear peptide design and not complete protein domains mimicking naturally folded HisRS present inside cells and in circulation. When analyzing autoantibody reactivity against linear epitopes, as performed in previous studies, there is a large risk of missing the detection of conformational-dependent autoantibodies. In addition, a previous study has discussed the importance of mapping of B cell responses over time to understand the epitope spreading and to allow for sub-grouping of heterogeneous diseases and correlation with disease activity [19]. The reactivity profile of anti-Jo1 antibodies against HisRS-FL, domains, and SV has so far only been assessed in sera and not in other biological samples such as the bronchoalveolar lavage fluid (BALF) and not in purified anti-Jo1 IgG which would limit the influence of other molecules that could interfere with the antigen binding in sera. In addition, only limited data is available concerning the behavior of anti-Jo1 antibody levels during the disease course and in relation to the different clinical phenotypes and treatments.

The findings described in previous studies have raised several important questions. Firstly, could anti-HisRS autoimmunity be initiated towards a specific region of the protein, e.g., WHEP domain which is highly expressed in the lungs [10], and during IIM/ASSD disease course spread throughout the HisRS molecule? Secondly, acknowledging the strong association between ILD and the anti-Jo1 response in IIM/ASSD [20], could anti-Jo1 autoantibodies targeting specific regions of HisRS be associated with distinct clinical phenotypes? Lastly, are anti-Jo1 autoantibodies in circulation recognizing the same HisRS epitopes as the autoantibodies found in the BALF of the lungs?

To address these questions, we extended previous epitope mapping studies to evaluate the reactivity profile of anti-Jo1 IgG and IgA from serum and BALF against HisRS-FL, the naturally occurring folded HisRS splice variant (SV), and separate HisRS domains (WHEP, CD, and ABD). Additionally, we explored the association between the anti-Jo1 reactivity to the full-length protein, single domains, and the splice variant of HisRS in relation to clinical manifestations in longitudinally collected serum samples and compared serum and BALF-derived anti-Jo1 autoantibodies collected at IIM/ASSD diagnosis. To get a deeper understanding of the binding profile and the development of the anti-Jo1 autoantibodies, we also investigated the affinity of these only against HisRS-FL at the time of disease diagnosis.

## Materials and methods

### Patient samples

Stored sera collected from consecutive patients with IIM/ASSD (19 anti-Jo1^+^ and 25 anti-Jo1^−^, cohort 1, Table 1) attending the Rheumatology clinic at Karolinska University Hospital, Stockholm, Sweden, between January 1, 1995, and June 30, 2017, were retrospectively identified for IgG purification. Samples from patients with incomplete clinical data for the purpose of the study were excluded. Classification of IIM was made according to the Bohan and Peter criteria [21, 22]. Griggs criteria [23] were applied for inclusion body myositis (IBM). The diagnosis of ASSD was based on the presence of anti-aaRS autoantibodies, plus one of the following features: ILD, myositis, arthritis, Raynaud’s phenomenon, fever, or mechanic’s hands [24]. The first available serum sample in relation to IIM/ASSD diagnosis was selected (median disease duration in Table 1). However, in three anti-Jo1^+^ and four anti-Jo1^−^ patients, the first available sera were collected at time points before diagnosis: specifically, up to 3 months before diagnosis, median −1 month (25–75th percentiles −3 to −1) for the anti-Jo1^+^ group, and up to 21 months before diagnosis, median −10 months (25–75th percentiles −19.5 to −4.25) for the anti-Jo1^−^ group. Longitudinal serum samples for IgG purification were available from 16 of the 19 anti-Jo1^+^ IIM/ASSD patients up to 24 years after diagnosis (Supplementary Fig. 2).

**Table 1:** Demographic data of cohort 1 at the time of first available serum sample*

	IIM/ASSD (***n***=44)	Anti-Jo1^**+**^ (***n***=19)	Anti-Jo1^**−**^ (***n***=25)
**Age**, mean years (SD)	57 (13)	52 (14)	61 (12)^a^
**Women**, *n* (%)	24 (55)	9 (47)	15 (60)
**Disease duration** in months, median (25–75th percentiles)^**^	0 (0–1)	1 (0–10)	0 (0–1)
**Anti-synthetase syndrome (ASSD)**, *n* (%)	28 (64)	19 (100)	9 (36)^b^
**Muscular manifestations**, *n* ever (%)			
Muscle weakness (pathological MMT8 and/or FI-2)	35 (83)	15 (79)	20 (87)
Muscle enzymes elevation (CK, LD, ASAT, ALAT)	35 (83)	15 (79)	20 (87)
Muscle inflammatory infiltrates	26 (62)	11 (58)	15 (65)
**Extra-muscular manifestations**, *n* ever (%)			
Interstitial lung disease (ILD)	25 (57)	16 (84)	9 (36)^c^
Skin rash^***^	14 (32)	5 (26)	9 (36)
Arthritis	18 (41)	11 (58)	7 (28)
Dysphagia	9 (21)	3 (16)	6 (24)
Raynaud’s phenomenon	2 (5)	2 (11)	0 (0)
**Smoking status**, *n* ever (%)	24 (55)	10 (53)	14 (56)
**Laboratory tests**			
CK, median μcat/L (25–75th percentiles)	4.3 (1.4–14.2)	3.8 (1.1–9.0)	4.4 (1.6–16.2)
CRP, median mg/L (25–75th percentiles)	4.0 (0.9–8.3)	7.0 (2.0–9.0)	2.0 (0.5–8.0)
**Autoantibodies**			
Positive anti-PL7, *n* (%)	2 (5.1)	0	2 (8.3)
Positive anti-PL12, *n* (%)	2 (5.1)	0	2 (8.3)
Positive anti-EJ, *n* (%)	1 (2.6)	0	1 (4.2)
Positive anti-OJ, *n* (%)	3 (7.7)	0	3 (12.5)
Positive anti-Mi-2, *n* (%)	3 (7.9)	1 (7.1)	2 (8.3)
Positive anti-SRP, *n* (%)	2 (5.1)	0	2 (8.3)
Positive anti-MDA5, *n* (%)	3 (7.9)	0	3 (12.5)
Positive anti-TIF1g, *n* (%)	3 (7.9)	0	3 (12.5)
Positive anti-SSA, *n* (%)	16 (36.4)	10 (52.6)	6 (24.0)
Positive anti-Ro52, *n* (%)	12 (38.7)	8 (47.1)	4 (28.6)
Positive anti-SSB, *n* (%)	0	0	0
Positive anti-U1 RNP, *n* (%)	5 (11.4)	2 (10.5)	3 (12.0)
Positive anti-Ku, *n* (%)	1 (2.5)	0	1 (4.0)
Positive anti-PmScl, *n* (%)	2 (4.9)	1 (6.3)	1 (4.0)
**Physician VAS**, median (25–75th percentiles)	40 (25– 60)	45 (32–60)	40 (17–50)
**Patient VAS**, median (25–75th percentiles)	40 (16–69)	44 (19–70)	32 (11–68)
**HAQ** (1–3), median (25–75th percentiles)	0.88 (0.00–1.50)	0.75 (0.19–1.25)	1.00 (0.00–1.63)
**MMT-8** (0–80), median (25–75th percentiles)	78 (67–80)	79 (77–80)	75 (64–80)
**Muscle activity score VAS**, median (25–75th percentiles)	15 (0 37.5)	4.5 (0–35.5)	15 (0–37.5)
**MDAAT**, median (25–75th percentiles)	0.07 (0.05–0.16)	0.12 (0.05–0.17)	0.06 (0.03–0.16)
**Extra-muscular activity,** median (25–75th percentiles)	32 (15–40)	40 (11–43)	24 (16–34)
**Immunosuppressive (IS) treatment**, *n* (%)			
No treatment	10 (26)	4 (25)	6 (26)
1 treatment	10 (26)	2 (13)	8 (35)
2 or 3 concomitant treatments	19 (49)	10 (63)	9 (39)
	**Healthy controls** (*n*=24)		
Age, mean years (SD)	59.3 (13.0)		
Women, *n* (%)	12 of 24 (50)		

*First available serum samples collected: (i) at diagnosis (0 months), sera were available from 6 anti-Jo1^+^ and 14 anti-Jo1^−^ patients; (ii) before diagnosis, sera were available from 3 anti-Jo1^+^ and 4 anti-Jo1^−^ patients (median months [25–75th percentile], −1 [−3 to −1] and −10[−19.5 to −4.25], respectively); (iii) after diagnosis, sera were available from 10 anti-Jo1^+^ and 7 anti-Jo1− patients (9 [1–99] and 1 [1–4], respectively).

*IIM* idiopathic inflammatory myopathies; *ASSD* anti-synthetase syndrome; *CK* creatinine kinase (reference values: 0.6–3.5 μkat/L); *CRP* C-reactive protein (0–3 mg/L); *VAS* visual analogue scale; *MDDAT* Myositis Disease Activity Assessment Tool; *HAQ* Health Assessment Questionnaire; *MMT-8* Manual Muscle Testing

1 treatment designates one of the following: methotrexate (Mtx), glucocorticoids (GC), intravenous immunoglobulin, or abatacept; 2 or 3 concomitant treatments designate all the possible following combinations: GC + azathioprine (Aza), GC + cyclophosphamide, GC + Mtx, GC + mycophenolate mofetil (MMF), GC + rituximab, GC + cyclophosphamide + rituximab, GC + Mtx + rituximab, or GC + MMF + rituximab

^**^Disease duration was calculated based on month and year of clinical diagnosis; ^***^Skin rash features: Periungual erythema, mechanic’s hand, Gottron’s sign, Gottron’s papules, V-sign, shawl sign, alopecia, erythroderma, periorbital edema, heliotrope rash

^a^*p* = 0.0236; ^b^*p* < 0.0001; ^c^*p* = 0.0020 vs anti-Jo1^+^ (Mann-Whitney’s test for quantitative variables and Fisher’s exact test for analysis of categorical variables were employed)

Consecutive patients with newly diagnosed IIM/ASSD were invited to perform bronchoscopy with BAL for research purpose between January 1, 2010, and December 31, 2016. Matching BALF and sera were available from the time of diagnosis from 10 patients who had given consent (6 anti-Jo1^+^ and 4 anti-Jo1^−^, cohort 2, Supplementary Table 1) and were retrospectively selected for this study. BALF samples were collected as previously described [25, 26]. Patients with suspicion of concomitant infection, malignancy, or respiratory failure not allowing to perform the bronchoscopy procedure were excluded.

Patients were defined as anti-Jo1^+^ if they had ever tested positive for anti-Jo1 antibodies by immunoprecipitation or line blot or ELISA immunoassays.

In cohorts 1 and 2, the mean age for the anti-Jo1^+^ IIM/ASSD group was lower compared to anti-Jo1^−^ IIM/ASSD (54 vs 61, *p* = 0.0467). All anti-Jo1^+^ patients from cohorts 1 and 2 (48% women) were diagnosed with ASSD, compared to 31% in anti-Jo1^−^ (*p* < 0.0001) and 88% of anti-Jo1^+^ IIM/ASSD had ILD in contrast to 34% in anti-Jo1^−^ patients (*p* < 0.0001). Demographics are presented in Table 1 and Supplementary Table 1. Serum samples from healthy control individuals (HC) were selected to match IIM/ASSD patients for age and gender (mean age 59 years, 50% women).

### Definition of clinical, laboratory and disease activity data

Signs of muscular involvement such as muscle weakness based on pathological manual muscle test-8 (MMT-8) with a total score < 80 and/or impaired muscle endurance evaluated by myositis functional index-2 (FI-2) [27], muscle enzyme elevation (creatine kinase (CK), lactate dehydrogenase (LD), aspartate aminotransferase (ASAT), alanine aminotransferase (ALAT)), and inflammatory infiltrates in muscle biopsies, present at any time during disease course, were recorded.

Extra-muscular manifestations such as ILD, arthritis, skin rash (periungual erythema, mechanic’s hand, Gottron’s sign, Gottron’s papules, V-sign, shawl sign, alopecia, erythroderma, periorbital edema, heliotrope rash), Raynaud’s phenomenon, and dysphagia present at any time during disease course were recorded. Smoking status was defined as never/ever smoker.

Diagnosis of ILD was based on the American Thoracic Society criteria [28]. All patients were screened with pulmonary function tests and high-resolution computer tomography (HRCT) of the lungs to confirm or exclude the presence of ILD with the exception of four anti-Jo1^−^ patients in cohort 1 who only underwent lung x-ray. In patients with ILD, spirometry test results (forced vital capacity (FVC), total lung capacity (TLC), and diffusion lung capacity of carbon monoxide (DLCO)) and HRCT data were retrieved at the time of each serum and BALF sample when available. The pattern of ILD (non-specific interstitial pneumonia (NSIP), usual interstitial pneumonia (UIP), and organizing pneumonia (OP)) was retrieved. When available, serial spirometry test results and HRCT reports made by experienced thorax radiologists were compared between the time of diagnosis and longitudinal time points. A 5–10% absolute increase or decline of predicted FVC and/or a 10–15% increase or decline of DLCO in combination with the evaluation of HRCT were considered to assign an ILD outcome as improvement, stable, or progression [29].

Longitudinal disease activity was assessed by prospectively collected variables of the IMACS (International Myositis Assessment & Clinical Studies group) disease activity core set measures [27] and by calculating the total improvement score according to the IMACS response criteria. For details, please see Supplementary Methods.

### ELISA and western blot analysis

Biotinylated HisRS variants and control proteins utilized for ELISA, western blot (WB), and affinity measurements were generated as previously described [30]. Information on antigen ID, molecular weight, and amino acid coverage of the proteins is depicted in Fig. 1A, Supplementary Table 2, and Supplementary Fig. 1. To avoid interference of other serum factors, IgG was purified from serum as described before [31, 32]. More information is found in Supplementary Fig. 2.

ELISA and WB experiments to evaluate the reactivity of serum and BALF-derived anti-Jo1 autoantibodies (IgG and IgA) against HisRS-FL, HisRS domains, and splice variant are described in Supplementary Methods. ELISA was executed in (i) IgG purified from serum of 44 IIM/ASSD and 24 HC individuals (anti-Jo1 IgG detection) and (ii) 13 BALF and 13 matched-sera, 10 from IIM/ASSD patients and 3 from HC. Total IgG, total IgA, anti-Jo1 IgG, and anti-Jo1 IgA were measured both in undiluted BALF and 1:500 diluted serum. The biotinylated variants of HisRS were added to streptavidin-coated plates in high excess compared to the amount of antibody tested to avoid the effects of different molar concentrations of antigen due to the different molecular weights of HisRS versions.

Anti-Jo1 IgG levels in serum (ng/mL) were calculated based on a standard curve generated from anti-Jo1 IgG enriched from a sera pool of 38 IIM/ASSD patients (Supplementary Fig. 3B). The antibody levels could be measured in the linear range between 5 and 100 ng/mL; therefore, a cut-off at 100 ng/mL was selected. These specific anti-Jo1 IgG were also enriched from serum by affinity chromatography as previously described, followed by a HisRS chromatography column [31, 32] (prepared in house, Supplementary Methods).

Autoantibody reactivity in IgG purified from serum of 19 anti-Jo1^+^ patients, 2 anti-Jo1^−^ patients, and 3 HC was also tested by WB (Fig. 1D, Supplementary Fig. 5B).

### Surface plasmon resonance

Affinity measurements of serum-derived IgG to HisRS-FL, close to diagnosis (between −0.25 and 0 years), from the 19 anti-Jo1^+^ patients were performed using surface plasmon resonance (SPR). The measurements were carried out using the Biacore T200 biosensor instrument (Cytiva), single cycle kinetics mode, and the Biacore T200 evaluation 3.1 software (Cytiva) was used for analyses. The measurements were done by capturing total IgG on the surface and flowing HisRS over the system to avoid measuring the avidity from the mix of polyclonal anti-Jo1 antibodies, for more details see Supplementary Methods.

### Statistical analysis

Continuous variables with normal distribution were presented as means with standard deviations (SD), while variables that violated normality were presented as medians with 25–75th percentiles [25-75th]. Comparison of categorical variables was performed using Fisher’s exact test or chi-square test, when appropriate. Friedman’s (followed by correction for multiple comparisons by Dunn’s test) or Mann-Whitney tests were employed when quantitative variables were compared among all groups or between two groups, respectively. Correlations between anti-Jo1 IgG/IgA reactivity levels and clinical data were performed using Spearman’s rank coefficient correlation. *p* < 0.05 denotes a significant difference. Data analysis was done using GraphPad Prism version 8 (La Jolla, USA). Multivariate modelling using principal component analysis (PCA) and orthogonal projections to latent structures discrimination analysis (OPLS-DA) was performed using SIMCA 15.0 (Umetrics, Sweden) following mean centering, log transformation, and UV scaling. Model performance was reported as the cumulative correlation R^2^X[cum], and predictive performance – as Q^2^[cum] based on seven-fold cross-validation.

## Results

### Reactivity profile against HisRS of serum and BALF-derived anti-Jo1 autoantibodies

#### Anti-Jo1 reactivity of IgG purified from the first available serum sample

Anti-Jo1 reactivity was evaluated by ELISA against HisRS (HisRS-FL), one HisRS splice variant (SV), and three HisRS domains (WHEP, CD, and ABD) (Fig. 1). In the first available serum sample, median 1 month [0–13] post-diagnosis, total IgG from anti-Jo1^+^ patients displayed stronger reactivity (although not statistically significant) against the HisRS-FL and the WHEP domain, in comparison with the CD and ABD, and splice variant SV (Fig. 1B, C, Supplementary Fig. 5A). The ELISA results were confirmed by WB (Fig. 1D, Supplementary Fig. 5B). Sixteen of 19 anti-Jo1^+^ patients showed reactivity to all HisRS antigens (with different degrees of binding). One patient (P4) presented exclusive binding to ABD and SV by ELISA and WB (Fig. 1B, D) but reactivity to HisRS-FL was only detected by WB (Fig. 1 D). Two patients (P1, P13) presented no reactivity against any of the HisRS antigens by ELISA and WB (Fig. 1, Supplementary Fig. 5). Together, these results confirm that anti-Jo1 antibodies recognize both conformation-dependent (ELISA) and conformation-independent epitopes (WB). Anti-Jo1^−^ and HC did not show reactivity towards HisRS-FL or any of the HisRS variant/domains (Fig. 1, Supplementary Fig. 5).

#### Anti-Jo1 reactivity of IgG and IgA present in BALF and matched serum samples

**Fig. 2:**
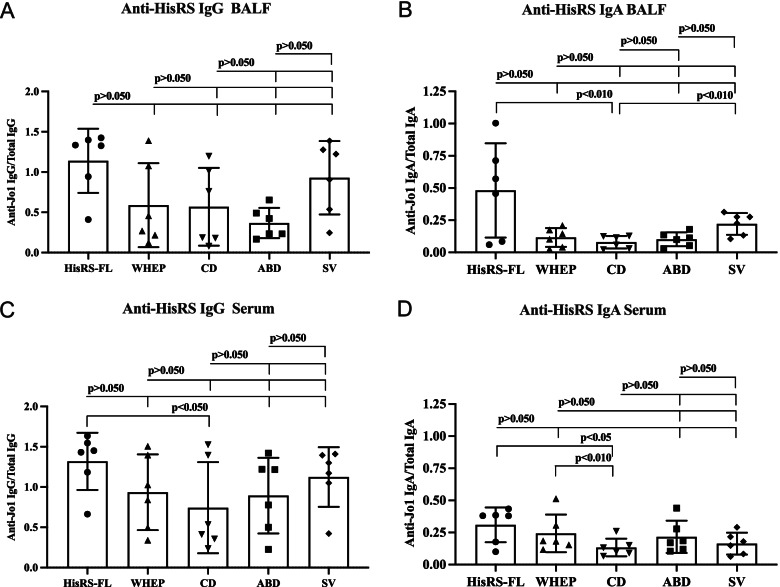
Reactivity of serum and BALF-derived anti-Jo1 antibodies. **A**, **C** Anti-Jo1 IgG and **B**, **D** IgA reactivity in BALF and paired serum were measured by ELISA in 6 anti-Jo1^+^ patients. Autoantibody levels were normalized to total values of IgG and IgA (*Y*-axis). Friedman’s tests corrected for multiple comparisons by Dunn’s test was applied. *p* < 0.05 was assumed as significantly different

In BALF, anti-Jo1 IgG and IgA displayed the strongest reactivity against HisRS-FL and SV in anti-Jo1^+^ IIM/ASSD patients (Fig. 2A, B). Similarly, in serum samples collected at the same time as the BALF, the highest IgG and IgA reactivity was found against HisRS-FL (Fig. 2C, D). In anti-Jo1^+^ IIM/ASSD patients, no anti-Jo1 autoantibody enrichment (frequency of anti-Jo1 antibody in total antibody amount) (*p* > 0.05) could be found in BALF in comparison to paired serum samples, for either IgG or IgA (Supplementary Fig. 4B, E).

BALF and paired sera from age/gender-matched HC and clinically diagnosed anti-Jo1^−^ patients did not display IgG or IgA reactivity against any of the variants (Supplementary Fig. 4).

#### Anti-Jo1 reactivity of IgG purified from serum of anti-Jo1^+^ patients collected longitudinally

**Fig. 3:**
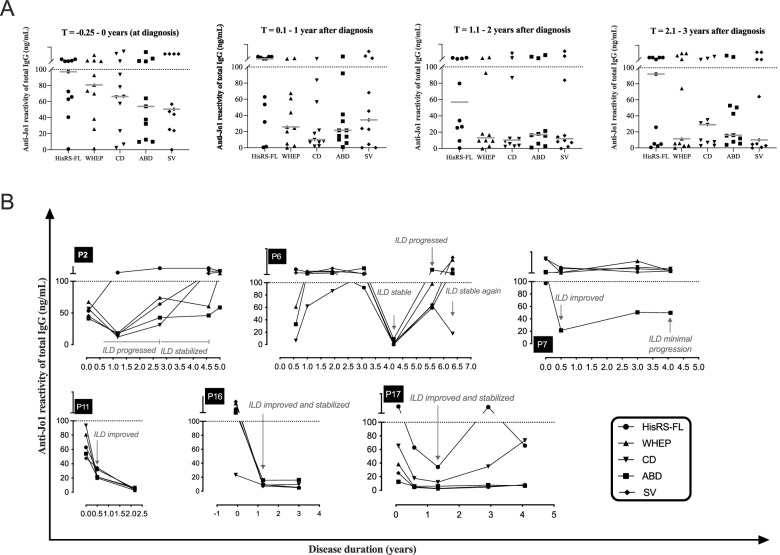
Reactivity of anti-Jo1 autoantibodies towards HisRS variant and domains decreases over time but remains high against HisRS-FL. **A** Reactivity against HisRS-FL, HisRS splice variant (SV), and HisRS domains (WHEP, CD, and ABD) displayed by total IgG purified from the first available anti-Jo1^+^ sera close to diagnosis (*T* = −0.25–0 years), *T* = 0.1–1, *T* = 1.1–2, and *T* = 2.1–3 years after diagnosis. Additional graphs displaying anti-Jo1 reactivity against HisRS-FL, variant, and domains are displayed in Supplementary Fig. 7 upper panel. **B** Anti-Jo1 reactivity of 6 anti-Jo1^+^ patients (P2, P6, P7, P11, P16, P17) displayed by total IgG purified from sera collected longitudinally. The *Y*-axis represents anti-Jo1 antibody levels against HisRS, measured in the total IgG fraction isolated from anti-Jo1^+^ IIM/ASSD sera. The X-axis represents disease duration in years. Gray italic sentences provide information on interstitial lung disease outcome during follow-up. Improvement, stabilization, or worsening of ILD was based on the comparison of spirometry test results (5–10% absolute increase or decline of predicted FVC and/or 10–15% increase or decline of DLCO) and of HRCT reports made by experienced thorax radiologists at the different time points. Concentration (ng/mL) of anti-Jo1 antibodies was calculated based on a standard curve derived from anti-Jo1 IgG isolated from a sera pool of 38 anti-Jo1^+^ IIM/ASSD individuals, and titers were measured in the linear range between 5 and 100 ng/mL (Supplementary Fig. 3B [32]). The letter P (Patient) followed by a number in each graph represents an anti-Jo1^+^ IIM/ASSD individual. Friedman’s tests corrected for multiple comparisons by Dunn’s test was applied in **A**. No significant differences were denoted

Longitudinal serum samples for IgG purification were available from 16/19 anti-Jo1^+^ IIM/ASSD patients. The highest reactivity levels of anti-HisRS-FL and anti-WHEP IgG were recorded at the time of diagnosis (median 97 and 81 ng/mL, respectively), in comparison to anti-CD, anti-ABD, and anti-SV IgG (median 66, 54, and 50 ng/mL, respectively) (Fig. 3A). Similar median IgG reactivity levels against HisRS were detected in the three anti-Jo1^+^ serum samples collected before diagnosis (median 100 ng/mL). Three years after diagnosis, median anti-HisRS-FL IgG reactivity levels were still almost as high as our limit of detection (median of 92 ng/mL), while the reactivity against WHEP and SV registered a decrease (median levels below limit of detection, Fig. 3A). The reactivity levels against CD and ABD decreased, remaining low thereafter (Fig. 3A).

The median antibody concentrations against the different HisRS antigens fluctuated over time and, in general, changed simultaneously. By following the longitudinal levels of anti-HisRS-FL antibodies, we noticed that the anti-HisRS-FL levels changed consistently with lung disease activity. In P11, P16, P17, P9, P10, P14, and P15, improvement or stabilization of ILD was registered when anti-HisRS-FL levels were lower than the levels recorded at the time of diagnosis (Fig. 3B, Supplementary Fig. 6). Accordingly, anti-HisRS-FL levels in P2 and P6 (Fig. 3B) increased in parallel to ILD progression. However, we also observed exceptions to this trend (Supplementary Fig. 6).

### Correlations between clinical data and reactivity profile

#### Reactivity of anti-Jo1^+^ IgG purified from first available serum in relation to clinical data

**Fig. 4:**
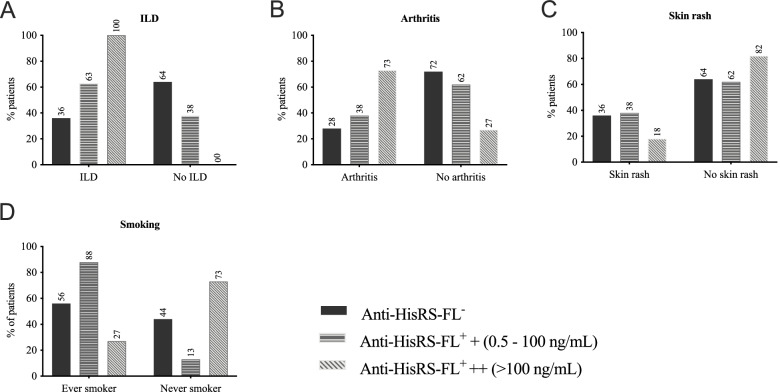
IIM/ASSD patients diagnosed with ILD and arthritis and less skin involvement harbor more reactive anti-Jo1 autoantibodies. **A–D** Percentage of IIM/ASSD patients distributed according to anti-HisRS full-length (HisRS-FL) reactivity levels, clinical diagnosis, and clinical manifestations. The anti-HisRS-FL reactivity displayed was measured in total anti-Jo1^+^ IgG purified from serum. Numbers on top of the bars represent the percentage of patients in the group. Anti-HisRS-FL^−^, anti-Jo1 IIM/ASSD-negative patients; anti-HisRS-FL^+^ +, anti-Jo1 IIM/ASSD-positive patients with low to moderate reactivity (0.5–100 ng/mL); anti-HisRS-FL^+^ ++, anti-Jo1 IIM/ASSD-positive patients with high HisRS reactivity (> 100 ng/mL). Antibody titers were calculated from OD (450 nm) using the standard curve in Supplementary Fig. 3B. IIM, idiopathic inflammatory myopathies; ILD, interstitial lung disease; ASSD, anti-synthetase syndrome. Statistical differences among groups are displayed in Supplementary Table 3

Considering the autoantibody levels targeting HisRS-FL in the first available sample close to the time of diagnosis, patients were stratified into low to moderate (*n* = 8, 0.5–100 ng/mL) or high anti-HisRS-FL reactivity (*n* = 11, >100 ng/mL, Fig. 4, Supplementary Table 3).

Muscle disease activity parameters did not statistically differ between those with low to moderate or high anti-HisRS-FL reactivity.

Anti-Jo1^+^ IIM/ASSD patients with high anti-HisRS-FL antibody levels were more likely to be diagnosed with ILD, ever through the disease course (100% compared to 63% for the anti-Jo1^+^ patient group with low to moderate anti-HisRS-FL IgG levels and 36% for anti-Jo1^−^ group, *p* < 0.05, Fig. 4A, Supplementary Table 3). ILD was present already at diagnosis in all anti-Jo1^+^ patients with reported lung manifestations. The pulmonary function (median values of FVC, TLC) in the low to moderate anti-HisRS-FL reactivity group was significantly lower compared to both anti-Jo1^+^ with high anti-HisRS-FL levels and anti-Jo1^−^ (51%, 67%, and 81% for FVC, and 54%, 70%, and 76% for TLC, in respective groups *p* < 0.05, Supplementary Table 3). Noteworthy, significantly more smokers were observed in the anti-Jo1^+^ group with low to moderate antibody levels (88%) compared to those with high anti-HisRS-FL IgG titers (27% *p* < 0.05, Fig. 4D).

The group with high anti-HisRS-FL antibody levels presented a higher percentage of arthritis (73%) in comparison to the low to moderate, and negative sub-groups (38% and 28%, respectively, *p* < 0.05, Fig. 4B, Supplementary Table 3). Anti-Jo1^+^ patients with low to moderate levels anti-HisRS-FL IgG levels, similarly to anti-Jo1^−^ patients, were more frequently diagnosed with skin manifestations (38%, 36%, and 18% for low to moderate, negative, and high anti-HisRS-FL response, respectively, *p* > 0.05, Fig. 4C, Supplementary Table 3).

Stratification based on high/low anti-WHEP, anti-CD, anti-ABD, and anti-SV IgG levels at the time of diagnosis largely reflected the observations described for HisRS-FL (data not shown).

#### Reactivity of BALF IgG/IgA close to IIM/ASSD diagnosis in relation to pulmonary function and BALF cellular content data

BALF levels of IgG anti-WHEP and anti-CD correlated negatively with several pulmonary function measures (VC, FVC, TLC, and FEV1, *p* < 0.05, *r* > −0.8810, Table 2). Anti-HisRS-FL IgA correlated negatively with FEV1 (*p* < 0.05, *r* > −0.7785), and anti-SV IgA correlated positively with FEV1_VC ratio (*p* = 0.044, *r* = +0.8407).

**Table 2:**
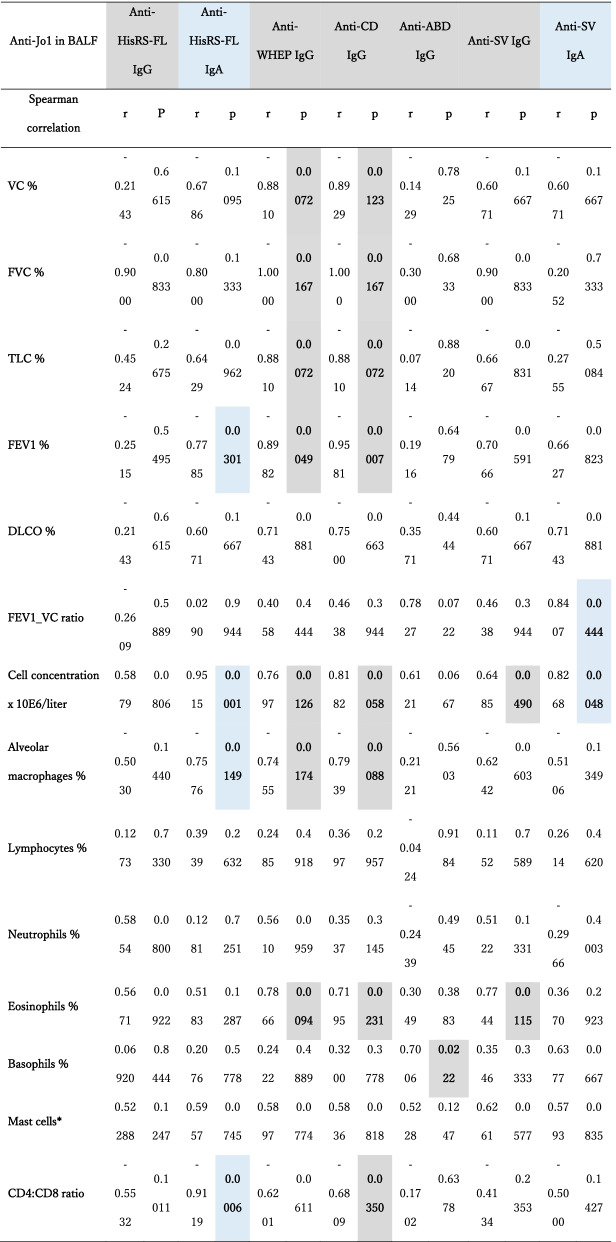
Correlations between clinical data and levels of anti-Jo1 IgG and IgA autoantibodies from BALF

Correlations between anti-Jo1 IgG and anti-Jo1 IgA reactivity levels and clinical data were performed using Spearman’s rank coefficient correlation with *p* two-tailed and 95% confidence interval. *p* < 0.05 denotes a significant difference

*Number of cells per high power field

*BALF* bronchoalveolar lavage fluid; *VC* vital capacity; *FVC* forced vital capacity; *TLC* total lung capacity; *FEV1* forced expiratory volume in 1 s; *DLCO* diffusion lung capacity for carbon monoxide

Correlations between IgG reactivities and BALF’s cellular content data are summarized in Table 2. Since the IgA reactivity in BALF against WHEP, CD, and ABD domains was very low, correlations were performed only with anti-HisRS-FL and anti-SV-IgA (Fig. 2B, Table 2).

### Multivariate data analysis

Multivariate data analysis was performed to identify correlations in the anti-HisRS reactivity profile and to obtain information on how this profile correlated with other clinical factors. Two types of principal component analysis (PCA) models were created: (1) including only the anti-HisRS reactivity data (described in Supplementary Results) and (2) including the anti-HisRS reactivity data combined with all other available information as described below.

**Fig. 5:**
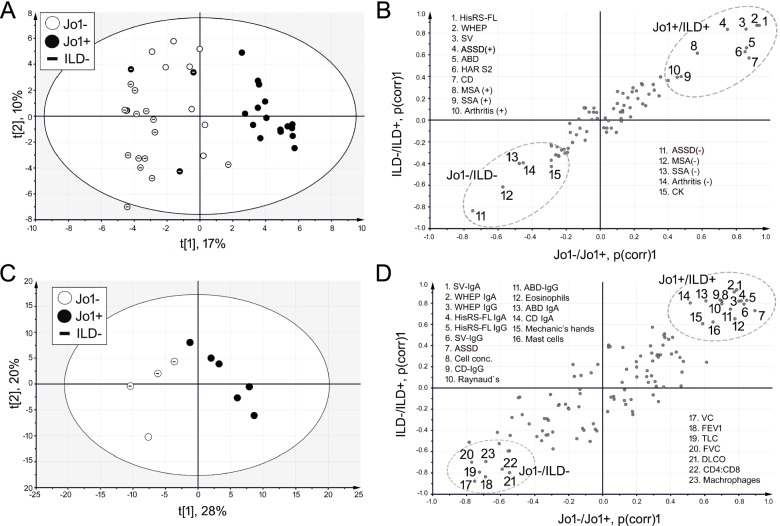
Multivariate data analysis shows a strong correlation between anti-Jo1 autoantibody reactivity profile, ILD and arthritis, and poor pulmonary function. **A** Principal component analysis (PCA) score plot of the baseline data obtained from the patients (cohort 1, *n* = 44) which demographics are listed in Table 1. **B** Shared and Unique Structures (SUS) plot obtained from two OPLS-DA models: (1) distinguishing the patients according to anti-Jo1 status (predictive component: *R*^2^ = 0.73, *Q*^2^ = 0.59) and (2) distinguishing the patients according to ILD status (predictive component: *R*^2^ = 0.74, *Q*^2^ = 0.60). The data processed is the same as in the PCA plot in cohort 1 (**A**). The factors that correlated most strongly with anti-Jo1^+^/ILD^+^ as well as anti-Jo1^−^/ILD^−^ are highlighted. **C** PCA score plot of the baseline data obtained from the patients (cohort 2, *n*=10) which demographics are listed in Supplementary Table 1. **D** Shared and Unique Structures (SUS) plot obtained from two OPLS-DA models: (1) distinguishing the patients according to Jo1 status (predictive component: *R*^2^=0.86, *Q*^2^=0.61) and (2) distinguishing the patients according to ILD status (predictive component: *R*^2^=0.76, *Q*^2^=0.43). The data processed is the same as in the PCA plot of cohort 2 in **C**

Anti-HisRS-FL reactivity data correlated strongly with ILD^+^ and anti-Jo1^+^ autoantibody status. Also, ASSD diagnosis, presence of MSAs, anti-SSA antibodies, and arthritis correlated with anti-Jo1^+^ and ILD^+^ patients in cohort 1 (Fig. 5A, B). We observed that ILD-negative patients (*n* = 19), independently of anti-Jo1 status, correlated negatively with anti-HisRS-FL and anti-WHEP reactivity (Fig. 5B). In cohort 2, which included fewer patients but where we had more information on pulmonary status, both the anti-HisRS-FL reactivity data from IgG and IgA as well as eosinophils and mast cells correlated strongly with anti-Jo1^+^ and ILD^+^ status (Fig. 5C, D). Inversely, higher levels of VC, FEV1, TLC, FVC, DLCO, CD4:CD8, and macrophages correlated prominently with anti-Jo1^−^ and ILD^−^.

### Affinity profile of anti-Jo1+ IgG purified from serum

**Fig. 6:**
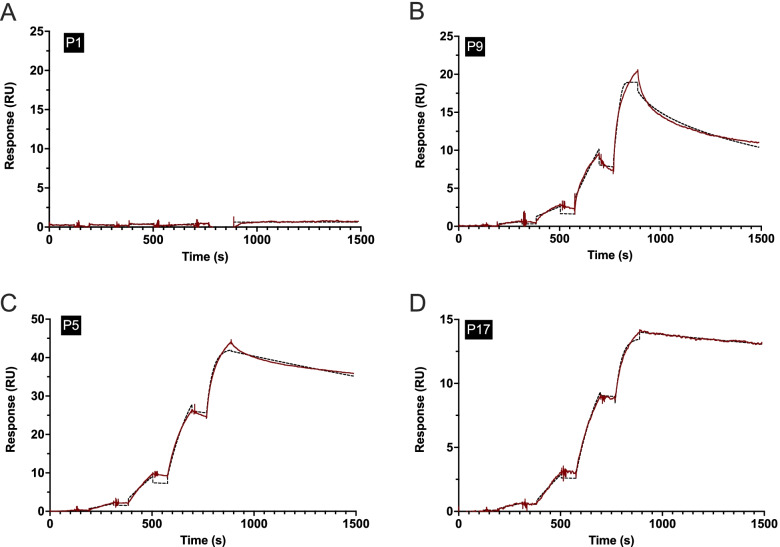
Affinity binding profiles against HisRS-FL for IgG purified from sera at first available sample close to diagnosis measured by SPR in single cycle kinetics mode. Sensorgrams showing the obtained signal in response units (RU, *y*-axis) over time (seconds, s, *x*-axis) from four representative patients (P1, P5, P9, and P17). **A** Three patients did not show any binding in SPR, as indicated by a flat line (same observation for P4 and P13, data not shown). **B** P9 is an example of a patient having a biphasic off-rate (^*Ave*^*k*_*d*_). This sort of heterogeneity was also observed for 6 other patients (P3, P7, P11, P12, P16, and P18). **C**, **D** A slower and more homogenous off-rate (^*Ave*^*k*_*d*_ < 0.0005 s-1) was observed for P5 and P17 (as well as P2, P8, P14, P15, P19), representing the other group (*n*=7) of patients. The biphasic binding profiles observed in approximately half the patients (*n*=7) could be explained by an avidity effect caused by the dimeric form of the antigen but can also be the result of the polyclonal nature of the patient sample and, thus, containing antibodies of different off-rates. Red lines represent the measured data values and dashed black lines represent the fit of the curve using the 1:1 Langmuir binding model. To distinguish the average values reported herein from traditionally reported affinity (*K*_*D*_) and dissociation rate constants (*k*_*d*_), we use the nomenclature ^*Ave*^*K*_*D*_ and ^*Ave*^*k*_*d*_. For control samples, see Supplementary material

The binding profiles of serum-derived IgG to HisRS-FL, close to diagnosis, from the 19 anti-Jo1^+^ patients were analyzed using surface plasmon resonance (SPR). Average kinetic constants could be determined for 14 of the patients, and in all cases, high average affinity profiles were observed (calculated ^Ave^*K*_D_ close to 1 nM). A selection of representative sensorgrams is shown in Fig. 6. IgG from patients P1, P4, and P13 did not show any binding to HisRS-FL in SPR, confirming the results from ELISA and WB, and P6 and P10 displayed too low responses for determination of kinetic constants. The remaining 14 patients could be divided into two groups based on the average affinity profile: one group (*n*=7) with a more biphasic off-rate (Fig. 6, as exemplified by P9) and another group (*n*=7) with a slower and more homogenous off-rate (Fig. 6, as exemplified by P5 and P17). Detailed analysis of the binding and fitting of the interaction to a suitable model is complicated due to several factors. Therefore, to distinguish the average values reported herein from traditionally reported affinity (*K*_*D*_) and dissociation rate constants (*k*_*d*_), we opted to use the nomenclature ^*Ave*^*K*_*D*_ and ^*Ave*^*k*_*d*_.

## Discussion

In the current study, we sought to understand (1) the response displayed by purified IgG anti-Jo1 autoantibodies derived from serum towards HisRS-FL, one HisRS splice variant, and single HisRS domains as well as IgG and IgA reactivity in paired serum and BALF towards the same protein variants; (2) possible associations between clinical manifestations and the pattern of anti-HisRS reactivity in circulation and in BALF both at the time of diagnosis and during the disease course; and (3) the affinity profile of anti-Jo1 autoantibodies against HisRS.

In our study, we could demonstrate that purified IgG anti-Jo1 autoantibodies in sera from a time close to diagnosis of IIM/ASSD exhibited high and multiple reactivities against the HisRS-FL, splice variant, and domains, with a particularly strong reactivity against the WHEP domain and the HisRS-FL. The ELISA results were confirmed by WB indicating that anti-Jo1 antibodies recognize both conformation-dependent (ELISA) and conformation-independent epitopes (WB). In BALF, the highest reactivity of both IgG and IgA anti-Jo1 autoantibodies was also directed towards the HisRS-FL.

Despite the overall similar antibody reactivity to the versions of the HisRS antigen seen in our patient cohort, we observed differences in clinical manifestations associated with the different levels of reactivities to the HisRS-FL antigen at the time of diagnosis. Thus, patients with high IgG serum levels towards HisRS-FL at diagnosis were more likely to ever present with ILD and arthritis, but less likely to have skin rash compared to patients with low to moderate anti-HisRS-FL IgG levels or anti-Jo1 negative. This is in agreement with a previous study where the levels of anti-Jo1 autoantibodies correlated with disease activity in different tissues/organs [33]. Furthermore, IgG anti-WHEP reactivity in BALF correlated with poor pulmonary function. These observations were further strengthened by applying an unbiased multivariate statistical analysis. The significant correlation between antibody reactivity (especially anti-WHEP) in the BALF at diagnosis and poor pulmonary function, together with BALF inflammatory content, supports the previously raised hypothesis of an association between autoantibody reactivity towards the WHEP domain and lung involvement in IIM/ASSD [34]. However, these results should be interpreted with caution, taken into consideration the low number of BALF samples and a possible selection bias as patients with more severe lung disease were excluded. Even though the WHEP domain appears to account for the main reactivity displayed by HisRS, our results suggest that antigenic regions other than the WHEP domain might be present [15] or develop at a later stage.

Thanks to the access to longitudinal serum samples and clinical data collected at the same time points, we could observe that, despite some exceptions, reactivity levels towards HisRS-FL changed over time consistently with the degree of lung disease activity. In fact, longitudinal levels of anti-HisRS-FL increased in line with ILD progression and decreased when recording an improvement of ILD. Notably, a high reactivity and high affinity against HisRS-FL early in the disease course remained high up to 3 years post-diagnosis despite treatment in some individuals which could explain why some patients with IIM/ASSD do not enter remission despite immunosuppressive treatment.

In addition to the ELISA experiments, we could confirm reactivity to the HisRS variants applying WB, indicating that anti-Jo1 antibodies could also recognize non-conformational epitopes within HisRS. All denatured HisRS fragments (non-conformational) were recognized by IgG anti-Jo1 antibodies with the exception of the CD, which could not be detected by WB suggesting that the reactivity towards CD is dependent on the 3-dimensional structure of the domain [17].

In this study, we have also developed a method to measure an average affinity of autoantibodies against HisRS-FL, using SPR. Strikingly, the majority of anti-Jo1^+^ patients presented anti-HisRS-FL antibodies with a high-affinity profile already at the time close to diagnosis. Recently, another study showed that individual anti-Jo1 monoclonal antibodies, selected based on somatic hypermutation using single-cell isolation and sequencing, also displayed affinities from low nM *K*_*D*_ and below [35]. Considering the generally high reactivity against all HisRS domains at diagnosis observed in this study, in combination with the fact that high-affinity autoantibodies were retrieved from patients with a recent diagnosis of IIM/ASSD, one can speculate that affinity maturation of anti-HisRS antibodies through somatic hypermutation together with epitope spreading has already happened before the onset of specific symptoms, ultimately leading to the diagnosis of IIM/ASSD. To confirm this hypothesis, further studies aiming at characterizing anti-Jo1 antibodies before clinical diagnosis are warranted.

The retrospective design is a limitation of our study as this may entail a selection bias in the available BAL fluids towards less severe lung involvement to be suitable for bronchoscopy. Another limitation is the definition of anti-Jo1 positivity based on results from the clinic from three different assays (immunoprecipitation, ELISA, line blot) which, despite their widespread use in research and clinical practice, still lack a standardization in large cohorts of myositis patients and, when compared, show varying agreement [36–38]. This could possibly explain why two selected anti-Jo1-positive patients presented no reactivity against any of the HisRS antigens by ELISA or WB. Another possibility for the discrepancy between the results acquired in the clinic and those obtained in this study may be the different time points of sampling as anti-Jo1 autoantibodies may disappear following low disease activity [33].

Another weakness of the study is the use of the Bohan and Peter criteria for classification of IIM as the EULAR/ACR classification criteria [39] for adult and juvenile IIM were not published at the time of patient inclusion. The low number of anti-Jo1^+^ patients represents another limitation of the study. However, since we aimed for an in-depth characterization of anti-Jo1 autoantibodies, the current study design was a feasible approach. Due to the previously discussed limitation of the small sample size, the statistical analysis using the non-parametric Friedman test, for comparison of IgG reactivity between the different HisRS variants (Fig. 1B, C), needs to be interpreted with caution. A strength of our study is that the antibody reactivities were analyzed in purified IgG which diminish interference of other factors in sera that could influence antigen binding. This was important since some of the reactivities detected using purified IgG could not be found when using sera. Even with the low number of patients included, the results from the paired serum-BALF analysis are consistent with a high IgG and IgA reactivity towards the HisRS-FL protein as well as towards the WHEP domain and SV at diagnosis (both in circulation and in the lung) as well as a solid report on the high-affinity profile of circulating IgG anti-Jo1 autoantibodies.

## Conclusions

In conclusion, anti-Jo1 autoantibodies of IgG and IgA subclasses from patients with IIM/ASSD bind multiple HisRS conformation-dependent and conformation-independent epitopes, already at the time of diagnosis of IIM/ASSD and both systemically and locally in the lung. We confirmed that the WHEP domain contains the major anti-Jo1 autoantibody epitope(s) which is strongly overrepresented among anti-Jo1 autoantibodies in circulation and BALF. These observations together with the correlation between high anti-HisRS-FL antibody levels in circulation and the presence of ILD support the previously raised hypothesis that the lung might be a site where aberrant immune activation against HisRS primarily occurs, leading to a systemic inflammatory condition, the anti-synthetase syndrome, with ILD as the main clinical manifestation.

## Supplementary Information

**Additional file 1.**

## Data Availability

All data generated or analyzed during this study are included in this published article and its supplementary information files.

## Abbreviations

aaRS: Aminoacyl transfer RNA synthetases
ABD: Anti-codon binding domain
ACPA: Anti-citrullinated protein/peptide antibodies
ASSD: Anti-synthetase syndrome
BALF: Broncheoalveolar lavage fluid
CD: Catalytic domain
CK: Creatinine kinase
CRP: Protein C-reactive
DAS-28: Disease activity score calculated for rheumatoid arthritis
DLCO: Diffusion lung capacity for carbon monoxide
DM: Dermatomyositis
ELISA: Enzyme-linked immunosorbent assay
FEV1: Forced expiratory volume in 1 s
FVC: Forced vital capacity
HAQ: Health assessment questionnaire
HC: Healthy controls
HisRS (Jo1): Histidyl-transfer RNA synthetase
HisRS-FL: Full-length histidyl-transfer RNA synthetase
HRCT: High-resolution computed tomography
IIM: Idiopathic inflammatory myopathies
IgG/A: Immunoglobulin G or A
IBM: Inclusion body myositis
ILD: Interstitial lung disease
Jo1^+^: Anti-Jo1-positive IIM/ASSD patients
Jo1^-^: Anti-Jo1-negative IIM/ASSD patients
MDDAT: Myositis disease activity assessment tool for extra-muscular global assessment
MMT-8: Manual muscle testing
NSIP: Non-specific interstitial pneumonia
OP: Organizing pneumonia
PM: Polymyositis
RA: Rheumatoid arthritis
SV: Splice variant
TLC: Total lung capacity
UIP: Usual interstitial pneumonia
VAS patient: Patient’s global disease activity assessment
VAS physician: Physician’s global disease activity assessment
VC: Vital capacity
WB: Western blot
