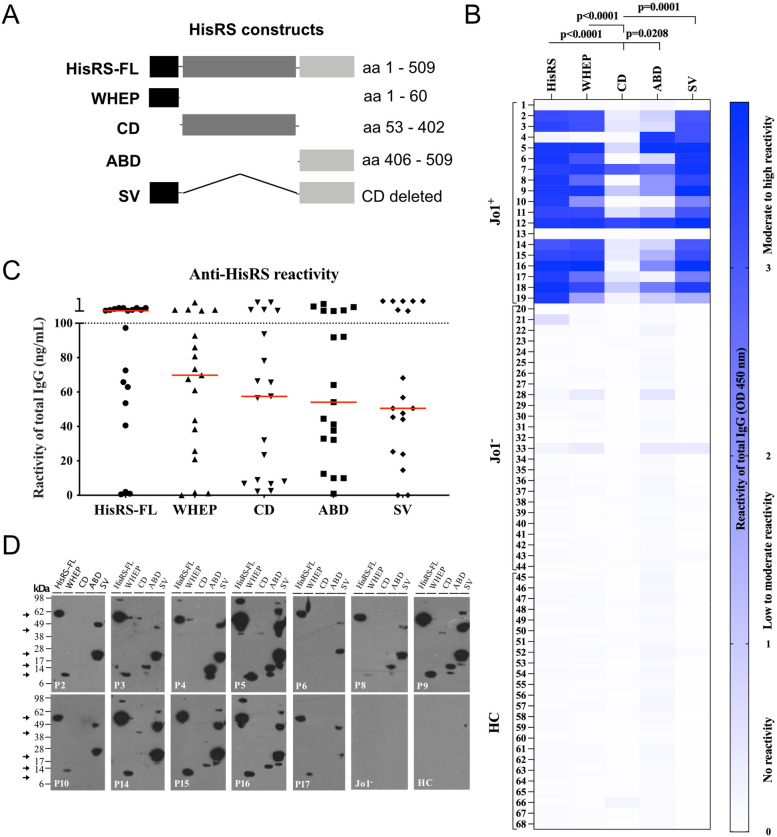# Correction: Longitudinal assessment of reactivity and affinity profile of anti-Jo1 autoantibodies to distinct HisRS domains and a splice variant in a cohort of patients with myositis and anti-synthetase syndrome

**DOI:** 10.1186/s13075-024-03446-y

**Published:** 2024-12-02

**Authors:** Antonella Notarnicola, Charlotta Preger, Susanna L. Lundström, Nuria Renard, Edvard Wigren, Eveline Van Gompel, Angeles S. Galindo-Feria, Helena Persson, Maryam Fathi, Johan Grunewald, Per-Johan Jakobsson, Susanne Gräslund, Ingrid E. Lundberg, Cátia Fernandes-Cerqueira

**Affiliations:** 1 Division of Rheumatology, Department of Medicine, Karolinska University Hospital, Karolinska Institutet, 171 64 Solna, Stockholm, Sweden; 2 Center for Molecular Medicine, Karolinska Institutet, Stockholm, Sweden; 3 Structural Genomics Consortium, Toronto, Canada; 4 Division of Physiological Chemistry I, Department of Medical Biochemistry and Biophysics, Karolinska Institutet, Solnavägen 9, 171 77 Stockholm, Sweden; 5 Laboratory of Tissue Homeosta- Sis and Disease, Skeletal Biology and Engineering Research Center, KULeuven, Louvain, Belgium; 6 Science for Life Laboratory, Drug Discovery and Develop- Ment, Stockholm, Sweden; 7 School of Engineering Sciences in Chemistry, Biotechnology and Health, Royal Institute of Technology (KTH), Stockholm, Sweden; 8 Department of Respiratory Medicine and Allergy, J7:30, Bioclinicum, Karolinska University Hospital, Karolinska Institutet, SE-171 76 Stockholm, Sweden; 9 4Dcell, 14 Rue de La Beaune, 93100 Montreuil, France

**Correction** **:** **Arthritis Res Ther 24, 62 (2022)**

**https://doi.org/10.1186/s13075-022-02745-6**

Following publication of the original article [1], we have recently received a comment by PubPeer that has found a mistake in our Figure 1 D. The western blot image uploaded for patient P16 was by mistake the same as for patient P15. It was only when putting together the image that the error occurred and all calculations, results and conclusions are thus unchanged. We have now updated figure 1 in the publication with the correct image.

Incorrect figure 1.


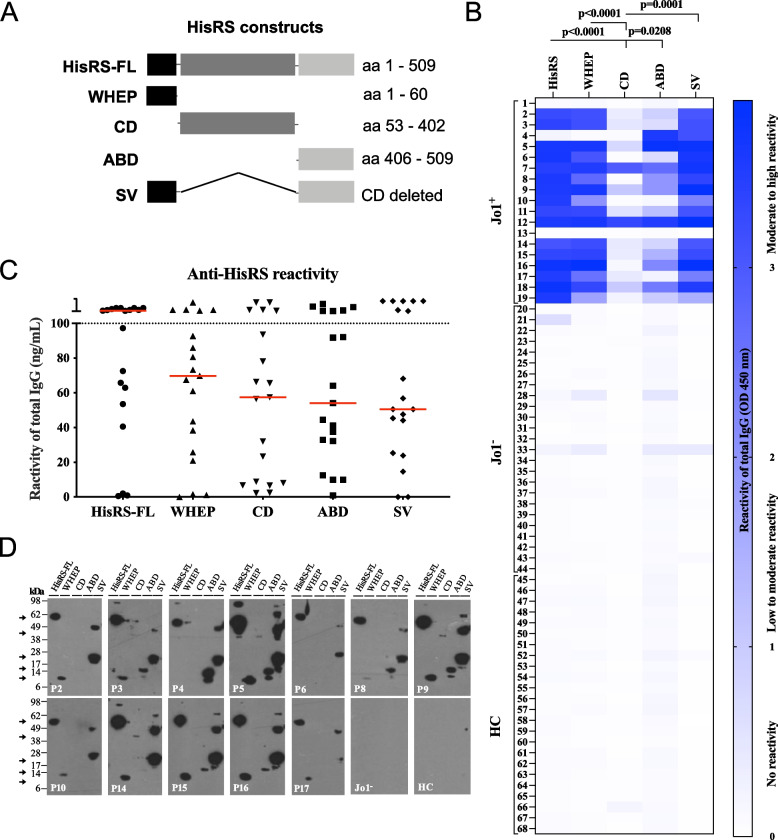


Correct figure 1.