# Supplementary material for: Longitudinal assessment of reactivity and affinity profile of anti-Jo1 autoantibodies to distinct HisRS domains and a splice variant in a cohort of patients with myositis and anti-synthetase syndrome
Source: Arthritis Res Ther. 2022 Mar 2;24:62. doi: 10.1186/s13075-022-02745-6 (PMC8889758; doi:10.1186/s13075-022-02745-6)
Supplement: Supplementary file 1 — Additional file 1. [file 13075_2022_2745_MOESM1_ESM.pdf]

SUPPLEMENTARY INFORMATION

Longitudinal assessment of reactivity and affinity profile of anti-Jo1 autoantibodies to distinct HisRS domains and a splice variant in a cohort of patients with myositis and anti-synthetase syndrome

Antonella Notarnicola*, Charlotta Preger*, Susanna L Lundström, Nuria Renard, Edvard Wigren, Eveline Van Gompel, Angeles Galindo Feria, Helena Persson, Maryam Fathi, Johan Grunewald, Per-Johan Jakobsson, Susanne Gräslund, Ingrid E Lundberg*, Cátia Fernandes-Cerqueira*. *Contributed equally

Supplementary Methods: Page 2 - 6

Supplementary Results: Page 7 - 8

Supplementary Table 1: Page 9 - 10

Supplementary Table 2: Page 11

Supplementary Table 3: Page 12 - 14

Supplementary Figure 1: Page 15

Supplementary Figure 2: Page 16

Supplementary Figure 3: Page 17 - 18

Supplementary Figure 4: Page 19- 20

Supplementary Figure 5: Page 21

Supplementary Figure 6: Page 22

Supplementary Figure 7: Page 23

Supplementary Figure 8: Page 24

Supplementary Figure 9: Page 25

Supplementary Figure 10: Page 26

References: Page 27-28

SUPPLEMENTARY METHODS

Definition of disease activity data

Variables of the IMACS (International Myositis Assessment & Clinical Studies group) disease activity core set measures (1), physician and patient global disease activity, CK, health assessment questionnaire (HAQ), MMT-8, muscle disease activity score and myositis disease activity assessment tool (MDAAT) encompassing constitutional, cutaneous, skeletal, gastrointestinal, pulmonary and cardiovascular disease scores, were retrieved within one month of diagnosis and at time of each longitudinal sampling from the Swedish Rheumatology Quality Register for IIM (Swemyonet) (2), and the Euromyositis register (3). The total improvement score was calculated according to the IMACS response criteria by comparing the IMACs core set measures at baseline and at the last available longitudinal sample. The total improvement score was used to define the response as minimal, moderate or major according to American College of Rheumatology (ACR response) (4). C-reactive protein (CRP) levels were collected within 1 month of diagnosis and at any time of longitudinal sampling as a proxy for disease activity.

ELISA assay for full-length, domains and variant of HisRS

Anti-Jo1 ELISA protocol was followed as described in (5) with slight modifications. Briefly, 384 well plates were coated with 25 ng of streptavidin (Sigma) overnight at 4°C, washed with PBS 0.05% Tween (PBST), and blocked with PBST 0.1% BSA for 1hour at room temperature (RT). Biotinylated recombinant HisRS antigens (HisRS-FL, WHEP domain, CD, ABD, and SV) diluted in PBST 0.1% BSA were added at 10 µg/mL and let to incubate in the plates for 1hour at RT. Patient samples tested in the ELISA (duplicates) were the following: i) IgG purified from serum at 10 µg/mL, for detection of anti-Jo1 IgG, ii) undiluted BALF, and iii) serum collected at the same time as the BALF and diluted 1:500. Total IgG, total IgA, anti-Jo1

IgG, and anti-Jo1 IgA were measured in BALF and matching serum samples. All samples were incubated in the plates for 2h at RT. Plates were then washed and incubated for 1h at RT with either 1:1000 diluted alkaline-phosphatase (ALP) monoclonal antibody MT78 (Mabtech) for IgG detection, or ALP monoclonal antibody MT20 (Mabtech) for IgA detection in BALF and serum. For anti-Jo1 reactivity analysis of total IgG, the peroxidase F(ab'2) fragment goat anti-Human IgG (Jackson Laboratories) diluted 1:10 000 in blocking buffer was employed (1h at RT). After the last washing step, pNPP substrate for ALP antibody detection or TMB for peroxidase antibody detection (Mabtech and Sigma) were added. The reaction with TMB was stopped after 15 minutes by adding 1M H_2SO_4. When using pNPP substrate, the OD was measured every 15 minutes at 405 nm and when using TMB substrate, the OD was measured one time at 450 nm (SoftMax Pro version 4.8, Molecular Devices).

IgG and IgA ELISAs followed instructions provided by the manufacture (Mabtech ref #3850-1AD-6 for IgG and 3860-1AD-6 for IgA).

ELISA control experiments

To evaluate whether we could detect the reactivity of anti-Jo1 IgG against HisRS variants we initially screened diluted serum (1:1000) and total IgG (10 µg/mL) enriched from the matching serum. Two representative patient samples (Patient 8 and 17) are displayed in Supplementary Figure 3. Although reactivity against HisRS-FL could be detected in serum, anti-WHEP and -ABD response could only be detected in the total IgG fraction. Therefore, the serum dilution utilized to test the reactivity against HisRS-FL, splice variants and domains in patients whom provided corresponding BALF samples was decreased to 1:500. Anti-Jo1 IgG purified from total IgG isolated from a sera pool of 38 IIM/ASSD patients was employed as a standard curve in order to calculate antibody levels and correlate with clinical data (5) (Supplementary Figure 3B). To be able to measure both low and high anti-Jo1 reactivities within the same dilution (10

μg/mL), the standard curve is only within the linear range between ≈5 and ≈100 ng/mL. Anti-Jo1 reactivity towards biotinylated and non-biotinylated HisRS-FL was compared to determine whether biotin interfered with the assay (Supplementary Figure 3C). Biotinylated HisRS-FL and non-biotinylated HisRS-FL were added to streptavidin pre-coated or high binding multiwell plates, respectively at a concentration of 1 μg/mL. The remaining protocol follows that described above.

Total IgG purified from serum from a healthy control, and Jo1 positive and negative patients were selected for control experiments to test anti-Jo1 reactivity against the background (PBS, streptavidin, or BSA coating, Supplementary Figure 3D), and unrelated protein (Supplementary Figure 3E, G) reactivity. In addition, a comparison was made between two versions of the same protein (HisRS-FL) with and without His_6 tag (Supplementary Figure 3F-G). Anti-Jo1 IgG selectively purified using the Jo1 affinity column (aJo1 IgG) were also employed for comparison. Two $Jo1^+$ (P23 and P25), one $Jo1^-$ (P30), and one HC BALF samples were also selected to run ELISA control experiment in a similar way as done for total IgG in circulation (Supplementary Figure 4B and E).

Western blot assay for full-length, domains and variant of HisRS

One microgram of each HisRS protein variant (HisRS-FL, WHEP, CD, ABD, and SV) was loaded on NuPAGE® Bis-Tris 4%-12% gradient gels under denaturing conditions and ran for 45 minutes at 200V in 2-(N-morpho-lino) ethanesulfonic acid (MES)-SDS antioxidant-containing buffer. Thereafter, proteins were transferred onto a nitrocellulose membrane (at 20V for 60 minutes in Glycine buffer) and blocked for 60 minutes with 5% milk in phosphate-buffered saline 0.05% Tween (blocking buffer). The different membranes, each containing a different HisRS protein variant, were individually incubated with anti-Jo1 IgG from 19 anti-$Jo1^+$ patients, 2 anti-$Jo1^-$ patients and 3 healthy controls (at 1 μg/mL diluted in blocking buffer).

Secondary antibody rabbit anti-human IgG HRP (diluted 1:1000 in blocking buffer, sc-2769 Santa-Cruz Biotechnology) was employed. Images were acquired and bands were quantified on a ChemiDoc XRS^{+} System using Image Lab™ Software (Bio-Rad). The correlation between ELISA and WB data reported in Supplementary Figure 5C was calculated by plotting OD 450 nm and mean intensity values, respectively (GraphPad Prism version 8).

Surface plasmon resonance

Affinity measurements were performed on serum-derived IgG from the 19 anti-Jo1^{+} patients using Biacore T200 biosensor instrument (Cytiva) and singe cycle kinetics. An anti-human Fab antibody (Cytiva) was covalently coupled to a Series S CM5 amine sensor chip (Cytiva) according to the manufacturer's instructions and used as capture molecule. Protein G purified IgG, diluted to a concentration of 5-10 µL/mL, were caught on the surface, followed by the addition of 0.08, 0.4, 2, 10, and 50 nM recombinant HisRS-FL at a flow rate of 30 µL/min. The anti-Fab antibody surface was regenerated with 10 mM Glycine-HCl pH 2.1. The experiment was performed at 25 °C using HBS-EP+ (Cytiva) with 1% BSA as running buffer. Response curves were obtained by subtracting a reference surface (no antibody captured) and a reference cycle (running buffer instead of HisRS-FL). The Biacore T200 evaluation 3.1 software (Cytiva) was used for analyses of the response curves and reaction rate kinetics calculated using the predefined 1:1 Langmuir binding model. Four anti-Jo1^{-} samples was selected as negative controls and run against HisRS in the same set-up as described above. In addition, the anti-Jo1^{+} IgG was also tested against another aminoacyl tRNA synthetase (ThrRS) as control protein. Two recombinant IgG, J-HARS-58 and L-TARS-11 (6), binding HisRS and ThrRS respectively, were used as positive controls. Representative sensorgrams of controls are presented in Supplementary Figure 10. ThrRS was produced as previously described (6). Of note, considering that the average affinity is the component being measured, there are some

facts to take into consideration; firstly, the analyzed total IgG samples are polyclonal, thereby likely containing a mixture of specific anti-Jo1 autoantibodies of different affinities, most likely present in very different concentrations. Here, an average affinity is measured, and the binding profile of an individual antibody clone within a polyclonal mix may be very different to what we report. For example, it is possible that high affinity antibodies in the sample mask low affinity antibodies. Secondly, the antigen HisRS-FL is a homodimer and this may give rise to an avidity effect, *i.e.* an apparent increase in affinity. Despite these complicating factors, the analysis was made using the predefined 1:1 Langmuir binding model to get comparable values among the patient samples.

SUPPLEMENTARY RESULTS

Total IgG purified from serum of anti-Jo1$^+$ patients collected longitudinally

Of the 19 anti-Jo1$^+$ patients included in this study, we could withdraw longitudinal sera from 16. However, not all of those 16 anti-Jo1$^+$ patients had longitudinal samples available at all and the same time-points. Therefore, we pooled the 7 anti-Jo1$^+$ individuals who provided sera both closer to diagnosis (from -0.25 years up to date of diagnosis) and in the consecutive years (up to 3 years post diagnosis, Supplementary Figure 7 upper panel). Similar to, and confirming the data displayed in Figure 3, Supplementary Figure 6 and 7, the median autoantibody levels targeting the different HisRS fragments consistently decreased from disease diagnosis and up to 3 years post diagnosis. The response to the HisRS-FL remained slightly higher in comparison to the SV splice variant as well as the CD and ABD WHEP domains (Supplementary Figure 7).

Anti-HisRS reactivity multivariate data analysis

The anti-HisRS reactivity data (HisRS-FL, WHEP, SV, CD, and ABD, described in Results section 1.1.), was subjected to PCA analysis (resulting in two components, R^2=0.89, Q^2=0.85). In the scores plot (shown in Supplementary Figure 8A) each dot represents the reactivity data obtained from a patient at a given time point. As expected, along the first component (x-axis), the patient data is separated according to anti-Jo1$^-$ vs anti-Jo1$^+$ patients and driven by all anti-HisRS reactivity data (independent of the HisRS specificity). Noteworthy, in addition to the anti-Jo1$^-$ patients (diamonds), the ILD negative patients (independent of anti-Jo1 status) also correlated negatively with anti-HisRS reactivity. Furthermore, the second component (y-axis), indicates that the ILD negative patients correlated negatively with HisRS-FL and WHEP. To investigate this further, two separate PCA models were created; one with CD and ABD (one component; R^2=0.82, Q^2=0.72, shown in Supplementary Figure 8B) and one with HisRS-FL

and WHEP (one component; R^2=0.88, Q^2=0.83, shown in Supplementary Figure 8C). From the plot in Supplementary Figure 8C, it is evident that HisRS-FL and WHEP are strongly associated with ILD status with a significant difference of p=2.9E-17 comparing ILD^+ and ILD^- anti-$Jo1^+$ patients. Interestingly, a similar trend can be observed for ILD^+ and ILD^- anti-$Jo1^-$ patients (p=1.5E-1). Compared to HisRS-FL and WHEP, the impact of ABD and CD on ILD status is less prominent for the anti-$Jo1^+$ patients (p=2.2E-4) and the trend is obviated (p=3.5E-1) for the anti-$Jo1^-$ patients, Supplementary Figure 8B.

Anti-HisRS reactivity and clinical information data analysis

Principal component analysis (PCA) analyses were performed on the first available sample obtained from the patients whose demographics are listed in Table 1 (cohort 1: 25 anti-$Jo1^-$ and 19 anti-$Jo1^+$ patients) as well as on the data obtained from the patients whose demographics are listed in Supplementary Table 1 (cohort 2: 4 anti-$Jo1^-$ and 6 anti-$Jo1^+$). Similar to the PCA scores plot of the anti-HisRS reactivity data (Supplementary Figure 8A), both models indicated a clustering of the anti-$Jo1^-$ patients and/or ILD^- patients (Figure 5A and 5C). In order to identify which factors correlated with anti-Jo1 and ILD status, two OPLS-DA models (per data set) were generated and plotted against each other (Figure 5B and 5D). As expected, the correlation between anti-$Jo1^-$ to anti-$Jo1^+$ and ILD^- to ILD^+ in both models is strongly positive (cohort 1: R^2=0.92 and cohort 2: R^2=0.76). In addition to the anti-HisRS reactivity data which strongly correlated with both anti-$Jo1^+$ and ILD^+, also ASSD, MSA, SSA and arthritis correlated with anti-$Jo1^+$ and ILD^+ in cohort 1 (Figure 5A and 5B).

SUPPLEMENTARY TABLES

Supplementary Table 1 Clinical and laboratory features, pulmonary function, and BALF characteristics from patients (cohort 2) who underwent BAL* at time of serum sampling.

	**Anti-Jo1^{+} (n=6)**	**Anti-Jo1^{-} (n=4)**
**Age**, mean years (SD)	60 (10)	58 (16)
**Women**, n (%)	3 (50)	3 (75)
**Anti-synthetase syndrome (ASSD), n (%)**	6 (100)	0 (0)
**Muscular manifestations, n ever (%)**		
Muscle weakness (pathological MMT8 and/or FI-2)	4 (67)	4 (100)
Muscle enzymes elevation (CK, LD, ASAT, ALAT)	3 (50)	4 (100)
Muscle inflammatory infiltrates	3 (50)	4 (100)
**Interstitial lung disease** (ILD), n (%)	6 (100)	1 (25)
**Skin manifestations**, n (%)		
Gottron's papules	0	2 (50)
Heliotrope rash	0	2 (50)
Shawl sign	0	2 (50)
Skin ulceration	0	1 (25)
V-sign	0	2 (50)
Mechanic's hands	3 (50)	0
**Raynaud's**, n (%)	3 (50)	0
**Dysphagia**, n (%)	1 (17)	1 (25)
**Arthritis**, n (%)	2 (33)	2 (50)
**Physician VAS**, median (25-75th percentiles)	48 (41 - 60)	65 (40 - 70)
**Patient VAS**, median (25-75th percentiles)	21 (11 - 76)	64 (30 - 97)
**MDAAT**, median (25-75th percentiles)	0.11 (0.06 - 0.35)	0.16 (0.11 - 0.20)
**HAQ**, median (25-75th percentiles)	0.65 (0.32 – 1.07)	0.07 (0.00 - 0.69)
**MMT-8**, median (25-75th percentiles)	80 (79 - 80)	79 (74 - 80)
**Muscle activity score VAS, median (25-75th percentiles)**	28 (0 - 55)	21 (20 - 21)
**CK**, median µcat/L (25-75th percentiles)	3.70 (1.15 - 16.05)	6,95 (3.60 - 12.78)
**CRP, median** mg/L (25-75th percentiles)	5.00 (2.00 - 7.00)	4.50 (2.25 - 6.00)
**Other autoantibodies**	SSA/RF/U1 RNP/PmScl/Ku	SSA/ACPA/RF/MDA5

**Immunosuppressive treatment**	3 no treatment; 1 GC/cycloph; 1 GC/MMF; 1 GC/cycloph/MMF/RTX	3 no treatment; 1 GC
**Smoking status**, n ever (%)	2 (33)	3 (75)
**HLA**	*03/*04; *03/*13; *03/*03; *01/*11; *01/*13; 1 DNA non-available	*09/*13; *04/*15; *03/*13; *04/*11
**VC**, median %	60	90
**FVC**, median %	55	91
**TLC**, median %	63	95
**FEV1**, median %	56	93
**DLCO**, median %	57	92
**FEV1_VC ratio**, median %	83	79
**Recovery**, median %	65	57
**Cell concentration x 10E6/liter**, median	423[a]	131
**Alveolar macrophages**, median %	68.05	82.75
**Lymphocytes**, median %	13.95	15.50
**Neutrophils**, median %	2.30	1.05
**Eosinophils**, median %	3.00[a]	0.10
**Basophils**, median %	0.00	0.00
**Mast cells**[**], median	17.50[a]	2.00
**CD4:CD8 ratio**, median	0.80	3.00

VAS (visual analogue scale) physician, physician's global disease activity assessment; VAS patient, patient's global disease activity assessment; MDDAT, Myositis Disease Activity Assessment Tool encompassing constitutional, cutaneous, skeletal, gastrointestinal, pulmonary and cardiovascular disease scores; HAQ, Health Assessment Questionnaire; MMT-8, Manual Muscle Testing; CK, Creatinine kinase; CRP, C-reactive Protein; GC, Glucocorticoids; Cycloph, Cyclophosphamide; MMF, Mycophenolate mofetil; RTX, Rituximab; HLA, Human leucocyte antigen; VC, Vital capacity; FVC, Forced vital capacity; TLC, Total lung capacity; FEV1, Forced expiratory volume in 1 second; DLCO, Diffusion lung capacity for carbon monoxide.

[*]BALF and matching serum were collected at diagnosis. [**]Number of cells per high power field;

[a]p<0,05 (Mann-Whitney's test).

Supplementary Table 2 Amino acid (aa) coverage and molecular weight of HisRS antigens and control proteins utilized in ELISA, WB, and affinity measurements.

ID	Antigen aa	Molecular weight (Da)
**Biotinylated antigens containing his tag**		
HisRS full-length (HisRS-FL, P12081*)	M1-C509	62485.0
WHEP domain	M1-K60	11687.3
Catalytic domain (CD)	K53-E402	43676.0
Anti-codon binding domain (ABD)	T406-C509	16906.3
Splice variant (WHEP+ABD, SV)	Δ K61-A398	24341.0
Interleukin 8 (IL-8, P10145*)	E21-S99	14183.1
Sacsin (Q9NZJ4*)	M1-K339	43348.8
IRF4 (Q15306*)	M1-E451	56715.8
**Biotinylated antigens without HisTag**		
HisRS full-length (HisRS-FL)	M1-C509	60019.4
WHEP domain	M1-K60	9221.6
Anti-codon binding domain (ABD)	T406-C509	14440.7

*Uniprot ID.

Supplementary Table 3 Demographics and clinical correlations with the anti-HisRS-FL reactivity profile at the first available sample taken close to the time of diagnosis. Patients are grouped according to anti-Jo1 status and anti-HisRS-FL IgG reactivity levels.

	**Anti-Jo1$^-$ (n=25)**	**Anti-Jo1$^+$ (n=19)**	**Low to moderate anti-HisRS-FL reactivity (n=8)**	**High anti-HisRS-FL reactivity (n=11)**	**P value (3 groups)**	**P value (2 groups)**
**Age**, mean years (SD)	61 (12)	52 (14)	53 (17)	52 (11)	0.060	0.885
**Women**, n (%)	15 (60)	9 (47)	2 (25)	7 (64)	0.175	0.168
**Disease duration**, median months (25-75th percentiles)	0 (0 - 1)	1 (0 - 10)	0 (0 - 6)	1 (0 - 52)	0.122	0.448
**Anti-synthetase syndrome (ASSD)**, n (%)	9 (36)	19 (100)	8 (100)	11 (100)	**<0.0001**	1.000
**Muscular manifestations**, n ever (%)						
Muscle weakness (pathological MMT8 and/or FI-2)	20 (87)	15 (79)	6 (75)	9 (82)	0.728	0.719
Muscle enzymes elevation (CK, LD, ASAT, ALAT)	20 (87)	15 (79)	6 (75)	9 (82)	0.728	0.719
Muscle inflammatory infiltrates	15 (65)	11 (58)	3 (38)	8 (73)	0.263	0.125
**Extra-muscular manifestations**, n ever (%)						
Interstitial Lung Disease (ILD)	9 (36)	16 (84)	5 (63)	11 (100)	**0.0016**	0.0580
Skin rash	9 (36)	5 (26)	3 (38)	2 (18)	0.532	0.603
Arthritis	7 (28)	11 (58)	3 (38)	8 (73)	**0.041**	0.181
Dysphagia	6 (24)	3 (16)	1 (13)	2 (18)	0.764	1.00
**Smoking status**, n ever (%)	14 (56)	10 (53)	7 (88)	3 (27)	**0.033**	**0.020**
**Laboratory tests**						
CK, median µcat/L (25-75th percentiles)	4.40 (1.60 - 16.20)	3.75 (1.13 - 9.03)	7.25 (1.78 - 40.20)	2.25 (1.00 - 5.88)	0.107	0.140

CRP, median mg/L (25-75th percentiles)	2.00 (0.50 - 8.00)	7.00 (2.00 - 9.00)	7.00 (2.00 - 10.00)	7.00 (2.50 - 7.88)	0.393	0.761
**Autoantibodies**						
Positive anti-SSA, n (%)	6 (24)	10 (53)	3 (38)	7 (64)	0.075	0.3698
Positive anti-Ro52, n (%)	4 (29)	8 (47)	1 (17)	7 (64)	0.0946	0.1312
**Physician VAS**, median (25-75th percentiles)	40 (17 - 50)	45 (32 - 60)	49 (32 - 75)	45 (33 - 58)	0.441	0.705
**Patient VAS**, median (25-75th percentiles)	32 (11 - 68)	44 (19 - 70)	45 (5 - 89)	44 (22 - 67)	0.829	0.899
**HAQ** (1–3), median (25-75th percentiles)	1.00 (0.00 - 1.63)	0.75 (0.19 - 1.25)	0.00 (0.00 - 1.75)	0.82 (0.44 - 0.97)	0.683	0.539
**MMT-8** (0–80), median (25-75th percentiles)	75 (64 - 80)	79 (77 - 80)	79 (66 - 80)	80 (77 - 80)	0.143	0.814
**Muscle activity score VAS**, median (25-75th percentiles)	15 (0 - 37.5)	4.5 (0 - 35.5)	19 (0 - 46)	0 (0 - 16)	0.155	0.210
**MDAAT**, median (25-75th percentiles)	0.06 (0.03 - 0.16)	0.12 (0.05 - 0.17)	0.13 (0.05 - 0.19)	0.10 (0.06 - 0.17)	0.682	0.970
**Extra-muscular activity**	24 (16 - 34)	40 (11 - 43)	20 (0 - 40)	41 (21 - 48)	0.308	0.400
**Immunosuppressive treatment**, n (%)					0.438	0.474
No treatment	6 (26)	4 (25)	2 (33)	2 (20)		
1 treatment	8 (35)	2 (13)	0 (0)	2 (20)		
2 or 3 concomitant treatments	9 (39)	10 (63)	4 (67)	6 (60)		
**Pulmonary function**						
FVC, median % (25-75th percentiles)	81 (17 - 107)	61 (52 - 76)	51 (50 - 52)	67 (61 - 86)	**0.006**	**0.024**
TLC, median % (25-75th percentiles)	76 (64 - 100)	63 (54 - 86)	54 (49 - 54)	70 (60 - 88)	**0.011**	**0.012**
DLCO, median % (25-75th percentiles)	67 (54 - 75)	58 (55 - 74)	57 (54 - 66)	71 (55 - 79)	0.520	0.339
HRCT, n pattern	5 NSIP; 1 OP; 3 UIP	11 NSIP; 3 OP; 2 UIP	5 NSIP; 1 OP; 1 UIP	6 NSIP; 2 OP; 1 UIP		

**Arthritis**						
DAS-28, median (25-75th percentiles)	4.74 (2.96 - 6.54)	4.56 (1.57 - 6.34)	6. 23	4.47 (1.56 - 5.69)	0.571	nd

CK, Creatinine kinase (reference values: 0.6 - 3.5 µkat/L); CRP, C-reactive Protein (0 - 3 mg/L); VAS (visual analogue scale) physician, Physician's global disease activity assessment; VAS patient, Patient's global disease activity assessment; MDDAT, Myositis Disease Activity Assessment Tool encompassing constitutional, cutaneous, skeletal, gastrointestinal, pulmonary and cardiovascular disease scores; HAQ, Health Assessment Questionnaire; MMT-8, Manual Muscle Testing; FVC, Forced Vital Capacity; TLC, Total Lung Capacity; DLCO, Diffusion Lung capacity for Carbon Monoxide; HRCT, High Resolution Computed Tomography; DAS-28, Disease activity score calculated for rheumatoid arthritis; NSIP, Non-Specific Interstitial Pneumonia; UIP, Usual Interstitial Pneumonia; OP, Organizing Pneumonia; nd, not determined.

Low to moderate reactivity (0.5 - 100 ng/mL) and moderate to high reactivity (>100 ng/mL) were calculated based on the anti-HisRS full length response displayed by total Jo1^{+} IgG purified from serum (Supplementary Figure 3B).

1 treatment designates one of the following treatments alone: methotrexate, glucocorticoids, intravenous Ig or abatacept; 2 or 3 concomitant treatments designate all the possible following combinations: glucocorticoids + azathioprine, glucocorticoids + cyclophosphamide, glucocorticoids + methotrexate, glucocorticoids + mycophenolate mofetil, glucocorticoids + rituximab, glucocorticoids + cyclophosphamide + rituximab, glucocorticoids + methotrexate + rituximab, or glucocorticoids + mycophenolate mofetil + rituximab.

Chi-square, Fisher's exact or Mann-Whitney's test were applied, when appropriate. P<0.05 denotes statistical significance. Reactivity was compared among anti-Jo1^{-}, low to moderate anti-HisRS-FL, and high anti-HisRS IgG levels (3 groups); or between low to moderate and high anti-HisRS IgG levels (2 groups).

*Definite, probable or possible myositis diagnosis according to Bohan and Peter's criteria.

Supplementary Figure 1

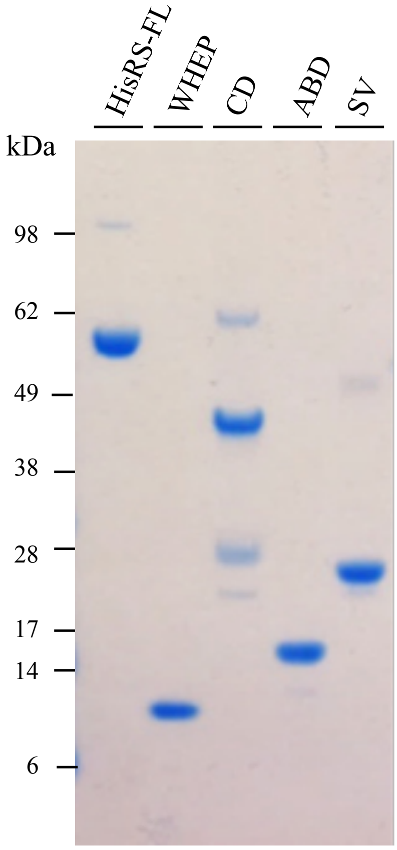

Supplementary Figure 1 Recombinant HisRS versions (schematic drawing in Figure 1) analyzed by SDS-PAGE and visualized by Coomassie blue staining.

Supplementary Figure 2

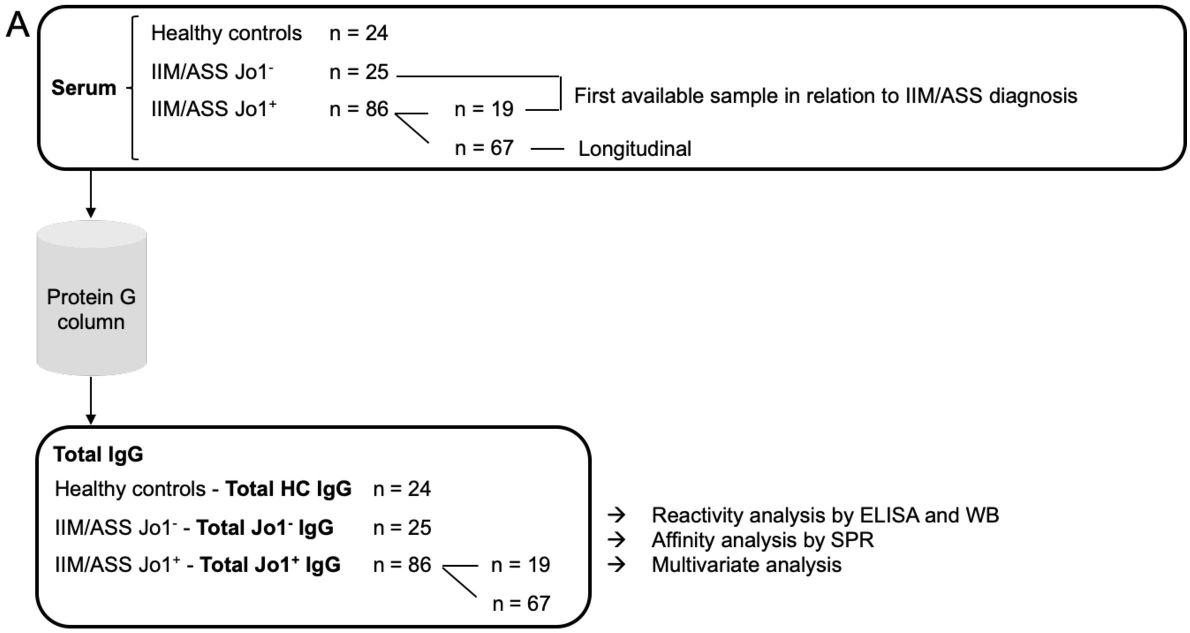

B

Number of anti-Jo1^{+} serum samples employed for total IgG purification from serum using the protein G column

**Patient ID code**	**Number of samples available**
P1	3
P2	5
P3	2
P4	1
P5	4
P6	7
P7	4
P8	13
P9	7
P10	2
P11	3
P12	2
P13	11
P14	4
P15	8
P16	3
P17	5
P18	1
P19	1
Total number of samples	86 (including 19 at diagnosis and 67 longitudinal)

Supplementary Figure 2 (A) Schematic illustration of total IgG purification process from serum (Scheme adapted from (5, 7)). (**B**) Description of number of serum samples available per patient (P) utilized for the individual purification of total IgG using a protein G column.

Supplementary Figure 3

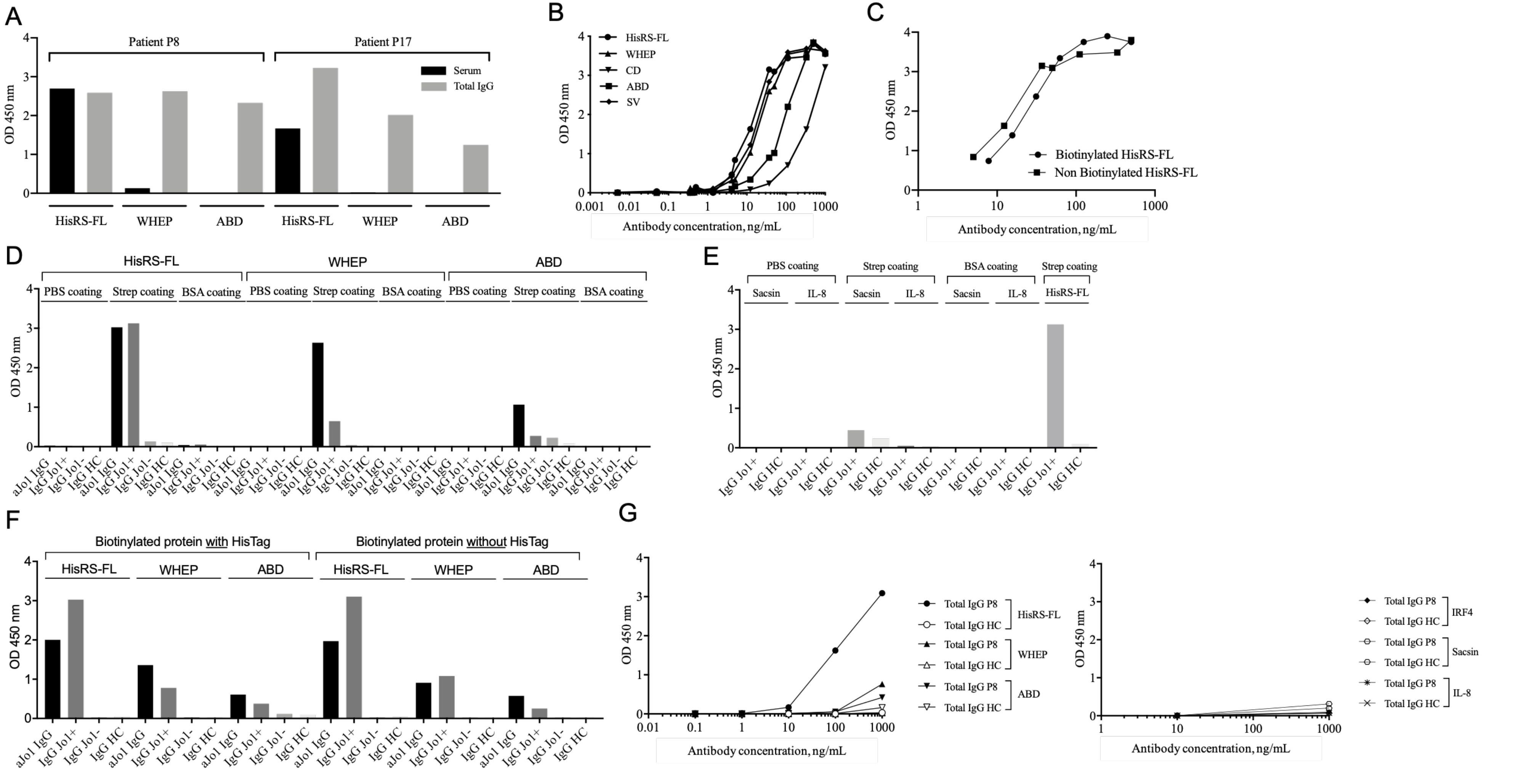

Supplementary Figure 3 Control experiments for anti-Jo1 ELISA optimization and validation. **(A)** ELISA to detect reactivity against HisRS-FL and two HisRS domains (WHEP domain and ABD) in serum (dilution 1:1000) and total IgG (10 μg/mL). **(B)** Standard curve (tested against HisRS-FL, HisRS domains and HisRS splice variants) designed utilizing anti-Jo1 IgG (aJo1 IgG) purified from a pool of serum collected from 38 $Jo1^+$ IIM/ASSD patients (5). **(C)** Similar anti-HisRS-FL reactivity is registered with either biotinylated or non-biotinylated HisRS-FL. **(D)** No anti-HisRS reactivity was found when using a coating solution (phosphate buffer, PBS or bovine serum albumin, BSA) other than streptavidin (Strep). **(E and G right panel)** Minimal reactivity was recorded against irrelevant proteins having molecular weight similar to HisRS variants (sacsin, IL-8 or IRF4, x axis represents total IgG concentration used in ng/mL). **(F)** No difference in anti-HisRS reactivity was observed when testing HisRS-FL, splice variant and domains with or without HisTag. **(G left panel)** Total IgG dilution curve to determine the optimal antibody concentration to be utilized in the ELISA for determination of reactivity against HisRS-FL and representative HisRS variants.

Supplementary Figure 4

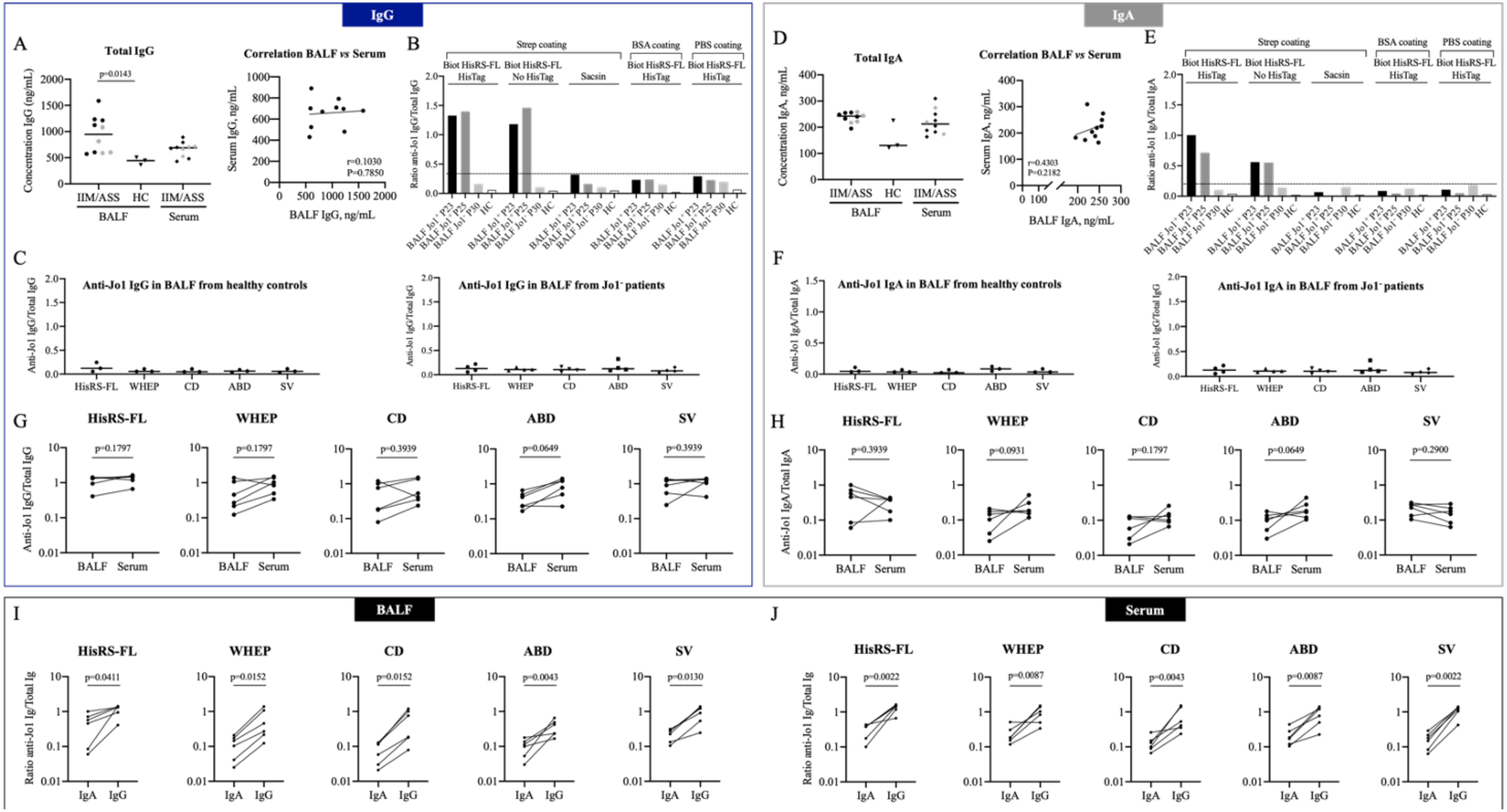

Supplementary Figure 4 (A) Total levels (ng/mL) and correlation between IgG in BALF and corresponding serum. **(B, E)** Control ELISA experiments showing minimal anti-Jo1 IgG and anti-Jo1 IgA reactivity in BALF against an irrelevant protein (sacsin), or when using PBS or BSA as a coating agent instead of streptavidin (strep). **(C and F)** Healthy control BALF IgG and IgA **(left panel)** and anti-Jo1$^-$ IIM/ASSD patients BALF IgG and IgA **(right panel)** do not present reactivity against HisRS-FL, WHEP domain, CD, ABD and SV. **(D)** Total levels (ng/mL) and correlation between IgA in BALF and corresponding serum**. (G)** IgG reactivity against HisRS-FL, WHEP domain, CD, ABD and SV is not enriched in BALF compared to corresponding serum in anti-Jo1$^+$ IIM/ASSD patients. **(H)** IgA reactivity against HisRS-FL, WHEP domain, CD, ABD and SV is not enriched in BALF compared to corresponding serum in anti-Jo1$^+$ IIM/ASSD patients. Comparison between anti-Jo1 IgG and IgA antibody levels in BALF **(I)** and serum **(J)**. In A and D, symbols in grey represent anti-Jo1$^-$ patients and symbols in black represent anti-Jo1$^+$ IIM/ASSD patients.

Supplementary Figure 5

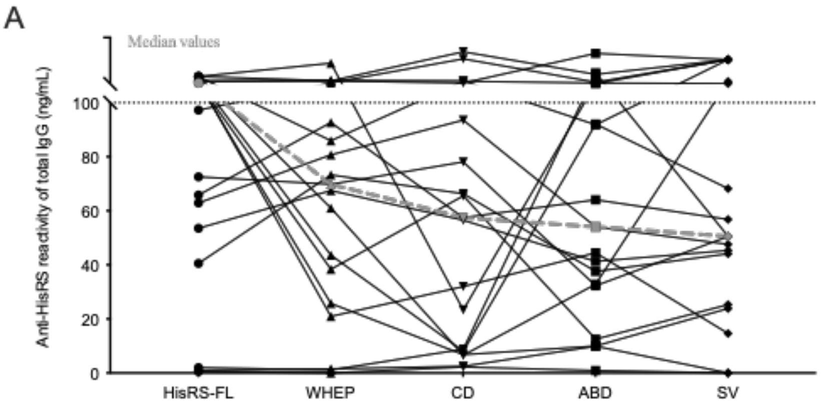

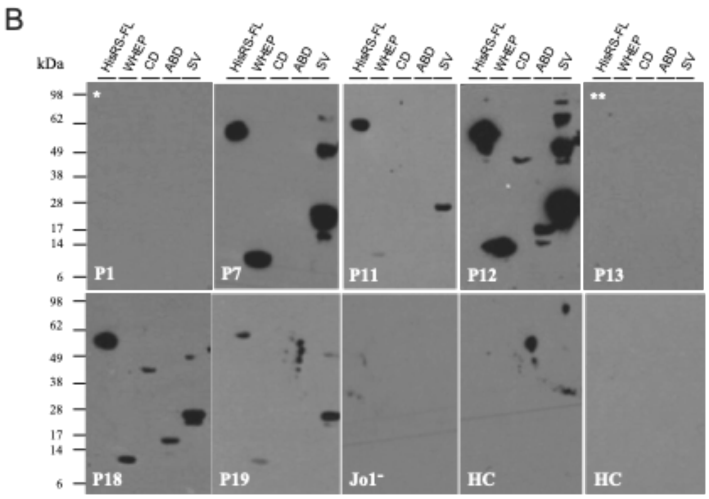

C

Correlation ELISA *vs* WB for total Jo1+ IgG

	r	p
HisRS-FL	0.7917	<0.0001
WHEP	0.8508	<0.0001
CD	0.6376	0.0033
ABD	0.5525	0.0014
SV	0.6105	0.0055

Supplementary Figure 5 Total Jo1$^+$ IgG response against HisRS-FL, WHEP, CD, ABD, and SV. **(A)** Graph represents individual anti-Jo1 IgG reactivity displayed by serum-derived total Jo1$^+$ IgG towards HisRS-FL, HisRS splice variant (SV), and HisRS domains (WHEP, CD, and ABD) measured by ELISA. **(B)** WB analysis of non-conformational HisRS epitopes recognized by total Jo1$^+$ IgG isolated from serum taken at different time points throughout IIM/ASSD disease. Antibodies from patients (P) 1, 7, 11, 12, 13, and 18 were collected between 0 and 4 years after diagnosis; Antibodies from patient P19 were collected 18 years after diagnosis. **(C)** Pearson correlation between anti-Jo1 reactivity accessed by ELISA and WB for serum-derived anti-Jo1 IgG against HisRS-FL, HisRS splice variant (SV), and HisRS domains (WHEP, CD, and ABD). p<0.05 denotes significant difference. P, patient; Jo1$^-$, anti-Jo1 negative IIM/ASSD patients; HC, healthy control. In B the asterisks * and ** represent patients P1 and P13 whom displayed negative anti-HisRS reactivity by ELISA.

Supplementary Figure 6

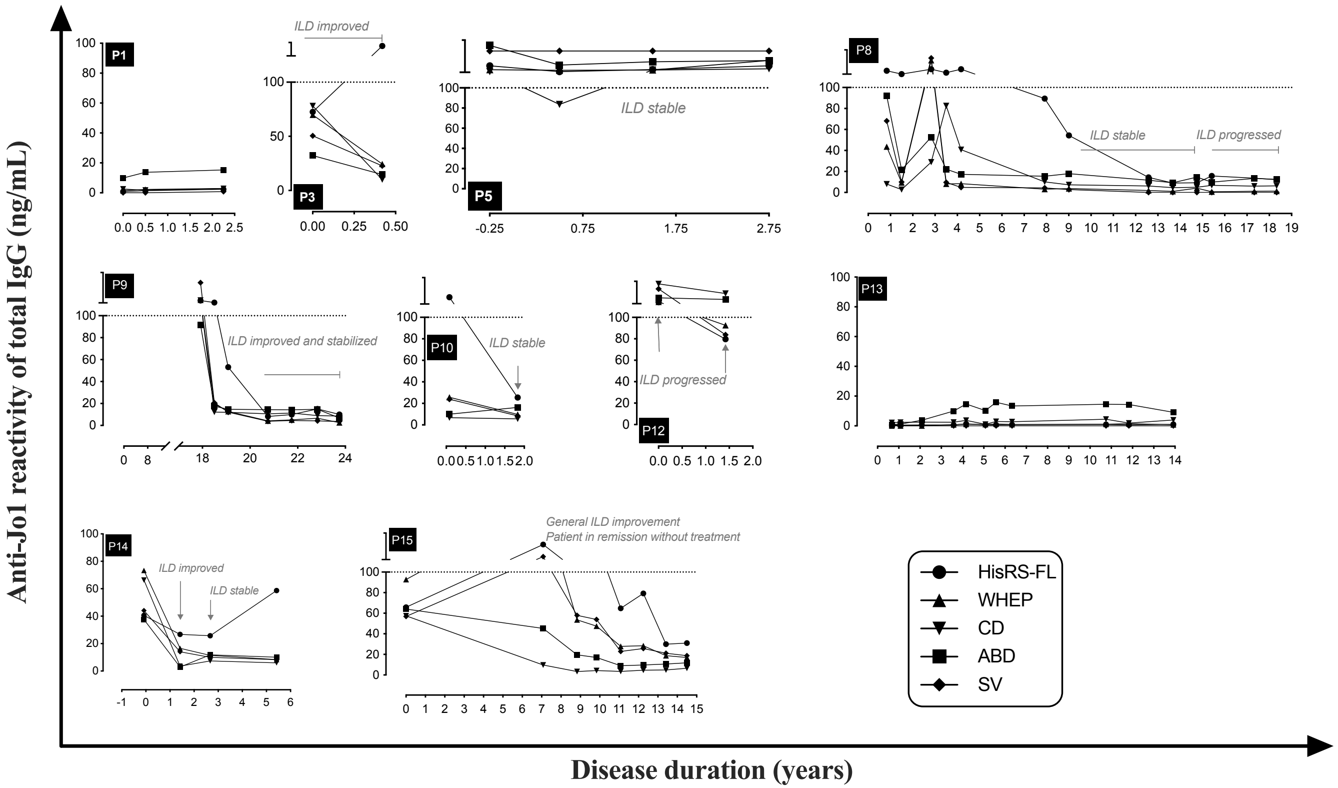

Supplementary Figure 6 Reactivity of anti-Jo1 autoantibodies towards HisRS variants over time for 10 of 16 anti-Jo1$^+$ patient. Anti-Jo1 reactivity of 10 anti-Jo1$^+$ patients displayed by total IgG purified from sera collected longitudinally. Y-axis represents anti-Jo1 antibody levels against HisRS, measured in the total IgG isolated from anti-Jo1$^+$ IIM/ASSD sera. X-axis represents disease duration in years, from the first available sample up to 24 years after diagnosis. Grey italic sentences provide information on rituximab introduction and/or follow-up on interstitial lung disease. Concentration (ng/mL) of anti-Jo1 antibodies was calculated based on a standard curve derived from anti-Jo1 IgG isolated from a sera pool of 38 anti-Jo1$^+$ IIM/ASSD individuals (Supplementary Figure 3B). Total anti-Jo1$^+$ IgG from different patients present distinct binding capacity to HisRS proteins. The letter P (Patient) followed by a number in each graph represents an anti-Jo1$^+$ IIM/ASSD individual.

Supplementary Figure 7

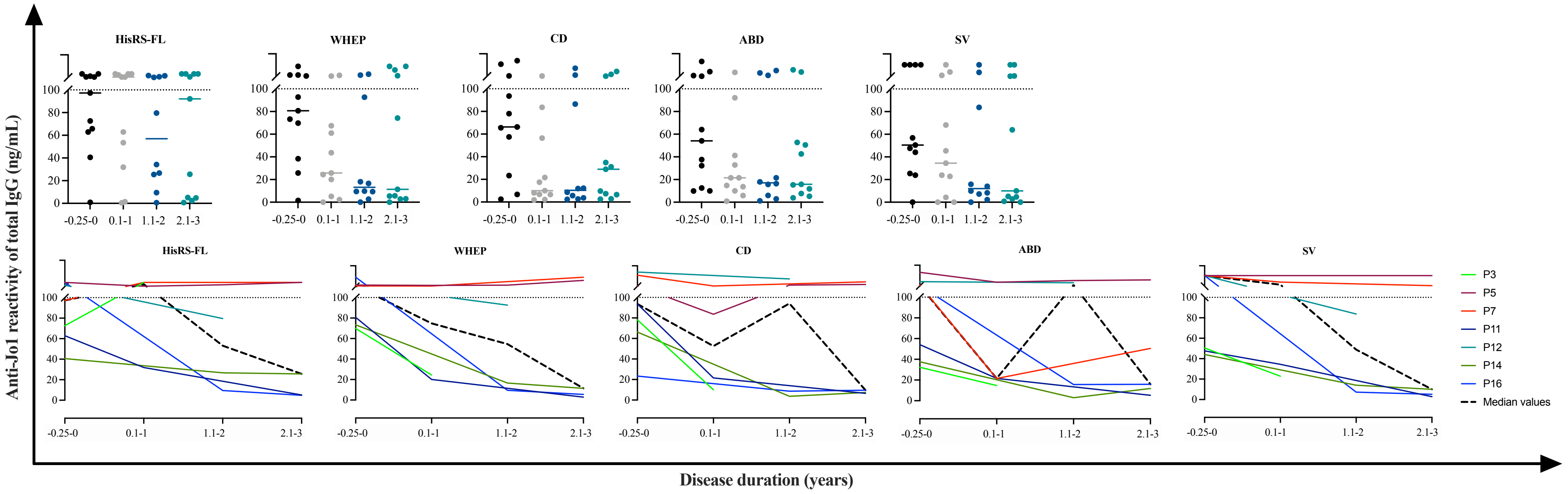

Supplementary Figure 7 (Upper panel) Reactivity (accessed by ELISA) against HisRS-FL, WHEP, CD, ABD and SV displayed by total IgG purified from sera at first available sample close to diagnosis (T = -0.25 – 0 years), and 0.1 – 1, 1.1 – 2, and 2.1 – 3 years after diagnosis. Since yearly IgG samples were not available from all 16 patients at follow-up, graphs in **(lower panel)** depict those patients (P3, P5, P7, P11, P12, P14, P16) from whom anti-HisRS reactivity (by ELISA) could be systematically accessed. Each colored line represents one patient (P).

Supplementary Figure 8

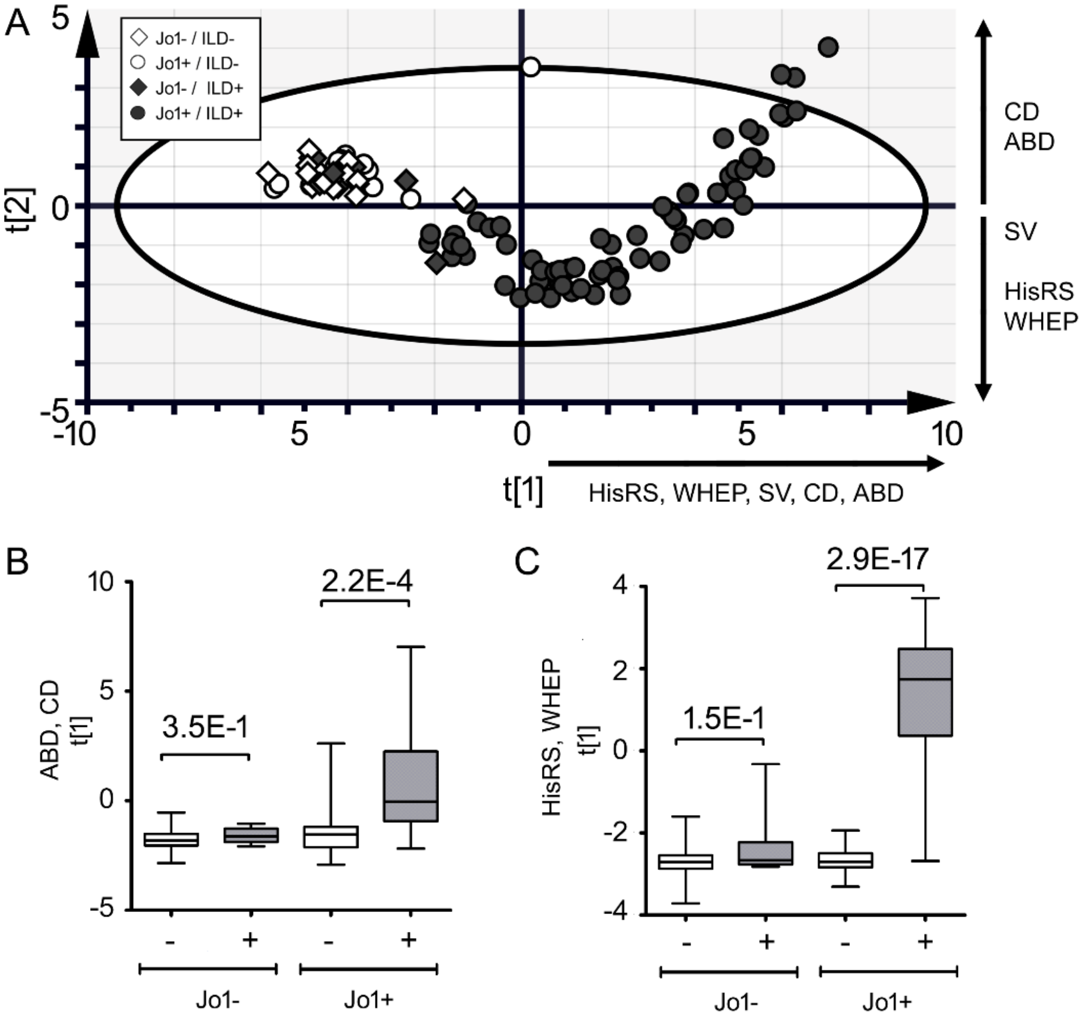

Supplementary Figure 8 (A) PCA scores plot of the anti-HisRS reactivity data (HisRS-FL, WHEP, SV, CD, and ABD). Each dot represents the reactivity data obtained from a patient at a given timepoint. The Jo1$^-$ (diamonds) and/or ILD$^-$ (white) patients cluster in one corner of the plot, indicating that their reactivity profiles are similar. Along the first component (x-axis), the separation between the profiles is driven by all anti-HisRS reactivity data. Along the second component (y-axis), the ILD$^-$ patients are negatively correlating prominently with HisRS-FL and WHEP. The loading plots of component 1 and component 2 are shown in Supplementary Figure 9**. (B)** The scores component of the PCA analysis of CD and ABD. The scores are presented as a box plot with minimum to maximum whiskers of the patient subgroups. **(C)** The scores component of the PCA analysis of HisRS-FL and WHEP. The scores are presented as a box plot with minimum to maximum whiskers of the patient subgroups. HisRS labelled in figure represents HisRS-FL.

Supplementary Figure 9

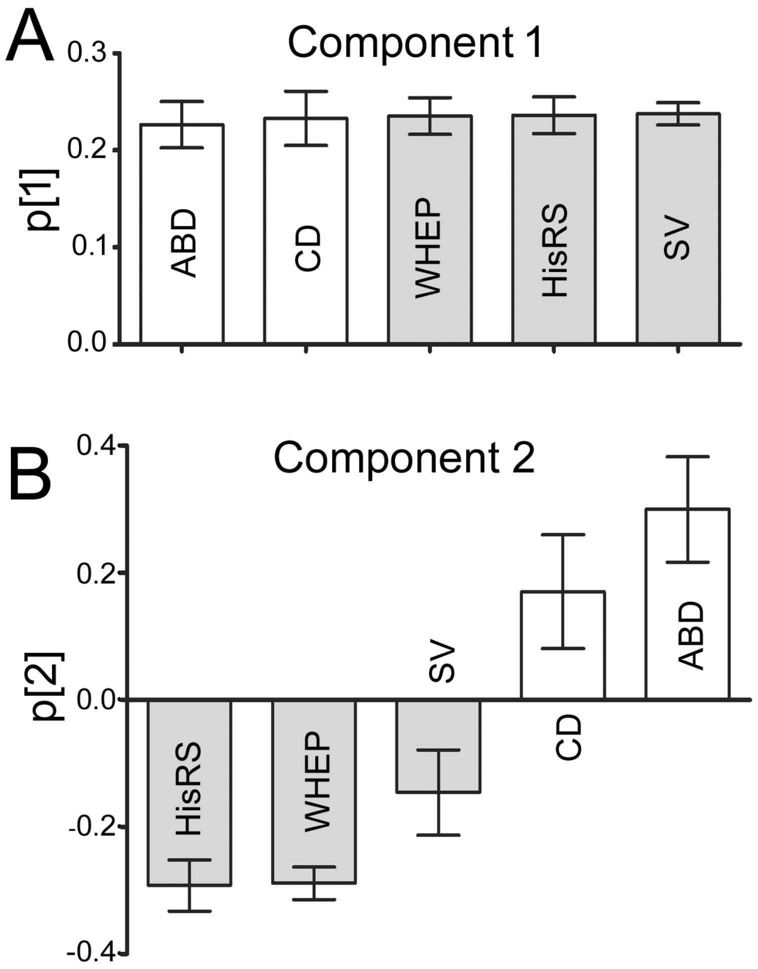

Supplementary Figure 9 Loading plots of the PCA analysis of the anti-HisRS reactivity data. **(A)** The correlation of the anti-HisRS reactivity data along the first component (x-axis in Supplementary Figure 8A). **(B)** The correlation of the anti-HisRS reactivity data along the second component (y-axis in Supplementary Figure 8A). Error bars represent 95% confidence intervals. HisRS labelled in figure represents HisRS-FL.

Supplementary Figure 10

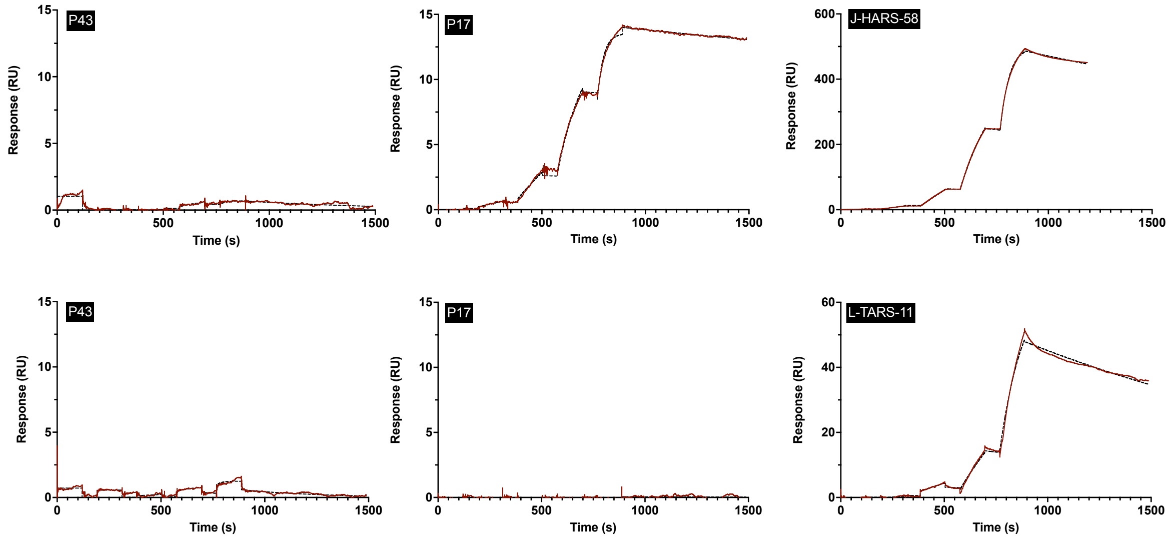

Supplementary Figure 10: Affinity binding profiles against HisRS-FL (top) and ThrRS (bottom) for IgG purified from sera at first available sample close to diagnosis or control recombinant IgG measured by SPR in single cycle kinetics mode. Sensorgrams showing the obtained signal in response units (RU, y-axis) over time (seconds, s, x-axis) from two representative patients; P43 (anti-Jo1-), P17 (anti-Jo1+), and two recombinant antibodies. P43 does not bind to either HisRS (top) nor ThrRS (bottom), as indicated by a flat line. P17 binds to HisRS (top), but not ThrRS (bottom). The recombinant IgG J-HARS-58 and L-TARS-11, specific for HisRS (top) and ThrRS (bottom) respectively, here used as positive controls. Red lines represent the measured data values and dashed black lines represent the fit of the curve using 1:1 Langmuir binding model.

REFERENCES

1. Rider LG, Werth VP, Huber AM, Alexanderson H, Rao AP, Ruperto N, et al. Measures of adult and juvenile dermatomyositis, polymyositis, and inclusion body myositis: Physician and Patient/Parent Global Activity, Manual Muscle Testing (MMT), Health Assessment Questionnaire (HAQ)/Childhood Health Assessment Questionnaire (C-HAQ), Childhood Myositis Assessment Scale (CMAS), Myositis Disease Activity Assessment Tool (MDAAT), Disease Activity Score (DAS), Short Form 36 (SF-36), Child Health Questionnaire (CHQ), physician global damage, Myositis Damage Index (MDI), Quantitative Muscle Testing (QMT), Myositis Functional Index-2 (FI-2), Myositis Activities Profile (MAP), Inclusion Body Myositis Functional Rating Scale (IBMFRS), Cutaneous Dermatomyositis Disease Area and Severity Index (CDASI), Cutaneous Assessment Tool (CAT), Dermatomyositis Skin Severity Index (DSSI), Skindex, and Dermatology Life Quality Index (DLQI). Arthritis Care Res (Hoboken). 2011;63 Suppl 11:S118-57.

2. Eriksson JK, Askling J, Arkema EV. The Swedish Rheumatology Quality Register: optimisation of rheumatic disease assessments using register-enriched data. Clinical and experimental rheumatology. 2014;32(5 Suppl 85):S.

3. Lilleker JB, Vencovsky J, Wang G, Wedderburn LR, Diederichsen LP, Schmidt J, et al. The EuroMyositis registry: an international collaborative tool to facilitate myositis research. Ann Rheum Dis. 2018;77(1):30-9.

4. Rider LG, Aggarwal R, Pistorio A, Bayat N, Erman B, Feldman BM, et al. 2016 American College of Rheumatology/European League Against Rheumatism Criteria for Minimal, Moderate, and Major Clinical Response in Juvenile Dermatomyositis: An International Myositis Assessment and Clinical Studies Group/Paediatric Rheumatology International Trials Organisation Collaborative Initiative. Arthritis Rheumatol. 2017;69(5):911-23.

5. Fernandes-Cerqueira C, Renard N, Notarnicola A, Wigren E, Graslund S, Zubarev RA, et al. Patients with anti-Jo1 antibodies display a characteristic IgG Fc-glycan profile which is further enhanced in anti-Jo1 autoantibodies. Sci Rep. 2018;8(1):17958.

6. Preger C, Wigren E, Ossipova E, Marks C, Lengqvist J, Hofstrom C, et al. Generation and validation of recombinant antibodies to study human aminoacyl-tRNA synthetases. J Biol Chem. 2020;295(41):13981-93.

7. Ossipova E, Cerqueira CF, Reed E, Kharlamova N, Israelsson L, Holmdahl R, et al. Affinity purified anti-citrullinated protein/peptide antibodies target antigens expressed in the rheumatoid joint. Arthritis Res Ther. 2014;16(4):R167.
